# Spectrum: fast density-aware spectral clustering for single and multi-omic data

**DOI:** 10.1093/bioinformatics/btz704

**Published:** 2019-09-10

**Authors:** Christopher R John, David Watson, Michael R Barnes, Costantino Pitzalis, Myles J Lewis

**Affiliations:** 1 Centre for Experimental Medicine and Rheumatology, William Harvey Research Institute, Bart’s and The London School of Medicine and Dentistry, Queen Mary University of London, London EC1M 6BQ, UK; 2 Oxford Internet Institute, University of Oxford, Oxford OX1 3JS, UK; 3 The Alan Turing Institute, London NW1 2DB, UK

## Abstract

**Motivation:**

Clustering patient omic data is integral to developing precision medicine because it allows the identification of disease subtypes. A current major challenge is the integration multi-omic data to identify a shared structure and reduce noise. Cluster analysis is also increasingly applied on single-omic data, for example, in single cell RNA-seq analysis for clustering the transcriptomes of individual cells. This technology has clinical implications. Our motivation was therefore to develop a flexible and effective spectral clustering tool for both single and multi-omic data.

**Results:**

We present Spectrum, a new spectral clustering method for complex omic data. Spectrum uses a self-tuning density-aware kernel we developed that enhances the similarity between points that share common nearest neighbours. It uses a tensor product graph data integration and diffusion procedure to reduce noise and reveal underlying structures. Spectrum contains a new method for finding the optimal number of clusters (*K*) involving eigenvector distribution analysis. Spectrum can automatically find *K* for both Gaussian and non-Gaussian structures. We demonstrate across 21 real expression datasets that Spectrum gives improved runtimes and better clustering results relative to other methods.

**Availability and implementation:**

Spectrum is available as an R software package from CRAN https://cran.r-project.org/web/packages/Spectrum/index.html.

**Supplementary information:**

Supplementary data are available at *Bioinformatics* online.

## 1 Introduction

Precision medicine is the concept that patients may be stratified into different subtypes to personalize therapy. A growing number of studies stratify patients using their genome-wide expression data (e.g. mRNA, miRNA, protein, methylation), such as those by The Cancer Genome Atlas (TCGA) (Agrawal *et al.*, 2014; Akbani *et al.*, 2015; Ceccarelli *et al.*, 2016; Ciriello *et al.*, 2015; Fishbein *et al.*, 2017; Network, 2013, 2014) and other consortia (Lefaudeux *et al.*, 2017). Clustering algorithms are used to find patient subtypes and may be run on data from single or multiple platforms. Single-omic cluster analysis is performed by algorithms such as: Monte Carlo consensus clustering (M3C) (John *et al.*, 2018), CLEST (Dudoit and Fridlyand, 2002), PINSPlus (Nguyen *et al.*, 2018) and similarity network fusion (SNF) (Wang *et al.*, 2014). However, clustering multi-omic data into an integrated solution is a major current challenge. State-of-the-art methods include: iClusterPlus (Shen *et al.*, 2009), SNF, CIMLR (Ramazzotti *et al.*, 2018) and PINSplus. The primary aims of multi-omic clustering are: (i) identifying a shared structure between platforms, and (ii) reducing noise from individual platforms. There is demand in this area for new, fast, effective methods and accessible software.

Single-cell RNA-seq is a technique that can be used to detect specific cell types by clustering of individual cell transcriptomes (Kiselev *et al.*, 2017). Analysing transcriptomes of individual cells may further our understanding of biology and has clinical applications. Single-cell RNA-seq data pose unique issues for clustering, as there are often more points to cluster and the data are usually found in dense globular clusters. Tools applied in this domain include: single-cell consensus clustering (SC3) (Kiselev *et al.*, 2017), Seurat (Butler *et al.*, 2018), MUDAN and single-cell interpretation via multikernel learning (SIMLR) (Wang *et al.*, 2017). Maintaining fast runtimes is important given the high number of points. SIMLR uses a sophisticated procedure to learn the optimal similarity matrix. However, it is very time consuming and it is not clear if SIMLR provides clustering performance advantages relative to other methods.

Spectral clustering refers to a class of algorithms that have become a hot topic in machine learning due to their ability to handle complex data (Ng *et al.*, 2002; Shu and Latecki, 2016; Xiang and Gong, 2008; Zelnik-Manor and Perona, 2005; Zhang *et al.*, 2011). They are characterized by clustering eigenvectors derived from a matrix representing the data’s graph (Ng *et al.*, 2002). Several of these methods are applied in genomic data analysis (Wang *et al.*, 2014, 2017). However, there have been a range of other developments in spectral clustering that provide ample opportunities for method development and implementation. A density-aware kernel (Zhang *et al.*, 2011) enhances local connections in higher density regions of the graph, however, this kernel does not self-tune to the data. Another method uses tensor product graph (TPG) integration and diffusion to integrate data sources and reduce noise (Shu and Latecki, 2016). One method retrieves eigenvectors of the data’s graph selected according to their multimodality for Gaussian mixture modelling (GMM) with the Bayesian Information Criterion (BIC) to decide on the optimal K (Xiang and Gong, 2008). The fast approximate spectral clustering (FASP) method (Yan *et al.*, 2009) enables rapid clustering of thousands of points on a desktop computer. Our aim was to assemble and advance this work.

Spectrum includes both methodological advancements and implements pre-existing techniques. Spectrum is distinct in a number of ways from previous spectral clustering-based tools (Wang *et al.*, 2014, 2017). Our contributions include: (i) a new self-tuning kernel that adapts to local density in the graph; (ii) a TPG data integration and diffusion procedure to combine different data sources and reduce noise; (iii) implementation of the FASP method for massive datasets; (iv) a new technique based on eigenvector distributional analysis to estimate the optimal *K*. Spectrum is provided as an accessible R software package (https://cran.r-project.org/web/packages/Spectrum/index.html) and has a detailed vignette.

## 2 Materials and methods

It is first instructive to describe the Zelnik-Manor self-tuning kernel (Zelnik-Manor and Perona, 2005) and the Zhang density-aware kernel (Zhang *et al.*, 2011) in order to understand our proposed kernel. The kernel is used to calculate the similarity matrix in spectral clustering that represents the data’s graph. Making a good similarity matrix in this initial step is key to getting good clustering performance.

### 2.1 Zelnik-Manor self-tuning kernel

The Zelnik-Manor kernel was designed to adapt to different data’s scale and so not require time-consuming parameter tuning. Let E denote an expression matrix $E\in R^{N\times M}$, where N is the number of points and M is the number of features, let A denote its similarity matrix $A\in R^{N\times N}$. Given a set of N points, $S=\left\{s_{1},\;s_{2},s_{3}\ldots ,s_{N}\right\}$, the Zelnik-Manor kernel is defined as:

$$A_{\mathrm{ij}}=\exp\left(\frac{-d^{2}(s_{i}s_{j})}{\sigma^{i}\sigma^{j}}\right)$$

Where $d\left(s_{i}s_{j}\right)$ denotes the Euclidean distance between points $s_{i}$ and $s_{j}$, $\sigma^{i}$ is a local scaling parameter and is calculated for every point $s_{i}$. $\sigma^{i}$ corresponds to $d\left(s_{i}s_{P}\right)$ where $s_{P}$ is the ${P\mathrm{th}}^{}$ nearest neighbour of $s_{i}$. $\sigma^{i}$ controls how rapidly $A_{\mathrm{ij}}$ falls off as $d\left(s_{i}s_{j}\right)$ increases. Selecting this parameter to equal one of the nearest-neighbour distances of $s_{i}$ allows the kernel to automatically tune to data with different scales. P is a free parameter and does not typically require parameter tuning to perform well.

### 2.2 Zhang density-aware kernel

Zhang *et al.* (2011) proposed a kernel that takes into account the common neighbours of points $s_{i}$ and $s_{j}$ when calculating $A_{\mathrm{ij}}$. The kernel increases $A_{\mathrm{ij}}$ when $s_{i}$ and $s_{j}$ share more neighbours, and therefore adapts to the data’s local density. Taking into account local density in this manner improves clustering performance by amplifying intra-cluster similarity (Zhang *et al.*, 2011).

$$A_{\mathrm{ij}}=\exp\left(\frac{-d^{2}(s_{i}s_{j})}{{2\sigma }^{2}(\mathrm{CNN}\left(s_{i}s_{j}\right)+1)}\right)$$

Where $\mathrm{CNN}\left(s_{i}s_{j}\right)$ is the number of points in the join region of the ε-neighbourhoods around points $s_{i}$ and $s_{j}$, where the ε-neighbourhood of a point represents the sphere around that point with radius ε. σ denotes a global scaling parameter.

In the Zhang kernel, both ε and σ must be tuned for each new dataset, unlike the Zelnik-Manor kernel that takes advantage of the nearest-neighbour distances to select σ quickly and gives good results. Parameter tuning for the Zhang kernel is slow and it is hard to define a good objective function. We propose a new kernel that adapts to local density in a similar way, but does not require parameter tuning for every new dataset. It does this by comparing the common nearest neighbours of points $s_{i}$ and $s_{j}$.

### 2.3 Adaptive density-aware kernel

We propose the following kernel:

(1) $$A_{\mathrm{ij}}=\exp\left(\frac{-d^{2}(s_{i}s_{j})}{\sigma^{i}\sigma^{j}(\mathrm{CNN}\left(s_{i}s_{j}\right)+1)}\right)$$

Where $\mathrm{CNN}(s_{i}s_{j})$ denotes the number of points in the intersection between the two sets of nearest neighbours of points $s_{i}$ and $s_{j}$. S is the free parameter that defines the number of nearest neighbours to include in each set. Because S is not a distance value like ε in the Zhang kernel it will automatically adapt to data with different scales.

Our kernel incorporates the advantages from both of the above kernels, whilst not requiring tuning to obtain good clustering performance. In this study, the kernel parameters were set to P=3 and S=7. These are free parameters, so there are not definitive values. Higher values will prefer global structures, while lower values local structures. However, these parameter settings were used for all experiments in this manuscript, including those on simulated data and are later shown to generalize well.

The first step of the Spectrum algorithm is to calculate the similarity matrix or matrices using the adaptive density-aware kernel. Note, this kernel is for continuous (non-binary) data. Additionally, if Spectrum is applied to multi-view data, the points must be matching between different views. If we are dealing with multiple datasets, then we have a set of T matrices $L=\left\{E_{1},E_{2},E_{3}\ldots ,E_{T}\right\}$, where $E_{i}\in R^{N\times M_{i}}$, $M_{i}$ is the number of features per sample from the ${i\mathrm{th}}^{}$ data type and N the number of points. Using Equation (1), in the case of multiple input datasets, this yields a new set of T similarity matrices, $Y=\left\{A_{1},A_{2},A_{3}\ldots ,A_{T}\right\}$, while if we have just one platform (T=1) we have a single similarity matrix A.

**Combining multi-view data and** **TPG** **diffusion.** For combining the similarity matrices (graphs) in the set Y, Spectrum uses a recent technique from the machine learning literature (Shu and Latecki, 2016) that involves calculating a cross view TPG from each pair of individual graphs. Cross-view TPGs capture higher order information of the data. The cross-view TPGs are integrated using linear combinations to form a single graph. Graph diffusion is then performed to reveal the underlying data structure. Shu *et al.* give a computationally efficient algorithm for this. The operation is mathematically analogous to the TPG approach but can be calculated using a non TPG which makes it much faster. Spectrum uses a minor modification of this method for a single data type. Note, the linear combination is weighted equally and assumes each view contributes towards a common structure, so very noisy data or data representing a very different structure should be excluded beforehand. The steps taken are as follows:

1. Combine similarity matrices from the set Y. If we are dealing with a single similarity matrix, T=1, then this step is skipped, but steps 2-5 are the same:
  (2) $$A=\sum_{i=1}^{T}A_{i}$$
2. Sparsify A by keeping only the ${Z\mathrm{th}}^{}$ nearest neighbours of each sample $s_{i}$ and setting the rest to 0. This makes a *k*-nearest-neighbour (*k*NN) graph. Let $R_{i}$ be the set of Z nearest neighbours for $s_{i}$, then:
  (3) $$A_{\mathrm{ij}}=\;\left\{\begin{array}{cc} A_{\mathrm{ij}} & j\in R_{i}\\ 0 & \mathrm{otherwise} \end{array}\right.$$
3. Row normalise A, so that each row sums to 1:
  (4) $$A_{\mathrm{ij}}=\;A_{\mathrm{ij}}/\sum_{j}A_{\mathrm{ij}}$$
4. Perform graph diffusion iterations. Let $Q^{1}=A$, and I be the identity matrix for A. Then for the ${t\mathrm{th}}^{}$ iteration from 2,…,iters:
  (5) $$Q^{t}=AQ^{t-1}A^{T}+I$$
5. We then take the final similarity matrix as $A^{*}=Q^{T}$. This ends the procedure. $A^{*}$ can now be used as the matrix for the rest of spectral clustering described in subsequent steps.

The parameters Z=10 and iters=5 are set in alignment with previous work that demonstrated their effective performance (Shu and Latecki, 2016). Shu *et al.* demonstrated that their algorithm is not very sensitive to these parameters.

**Spectral clustering of similarity matrix.** Starting with $A^{*}$, Spectrum uses the Ng spectral clustering method (Ng *et al.*, 2002), but with the eigengap heuristic to estimate the number of clusters and GMM to cluster the final eigenvector matrix. More specifically:

1. Using D, the diagonal matrix whose $\left(i,i\right)$ element is the sum of $A^{*}$’s ${i\mathrm{th}}^{}$ row, construct the normalized graph Laplacian L:
  (6) $$L=D^{-1/2}A^{*}D^{-1/2}$$
2. Perform the eigendecomposition of L and thus extract its eigenvectors $x_{1},\;x_{2},\;\ldots \;x_{N}$ and eigenvalues $\lambda_{1},\lambda_{2}\ldots \lambda_{N+1}$.
3. Evaluate the eigengap for eigenvalues, starting with the second eigenvalue, n=2, and choose the optimal k, denoted by $k^{*}$, for which the eigengap is maximised:
  (7) $$k^{*}=\underset{n}{\mathrm{argmax}}\left(\lambda_{n}-\lambda_{n+1}\right)$$
4. Get the $x_{1},\;x_{2},\;\ldots \;x_{k^{*}}$, $k^{*}$ largest eigenvectors of L, then form the matrix, $X=\left[x_{1},\;x_{2},\;\ldots \;x_{k^{*}}\right]\in R^{N\times k^{*}}$ by stacking the eigenvectors in columns.
5. Form the matrix Y from X by renormalizing each of X’s rows to have unit length:
  (8) $$Y_{\mathrm{ij}}=\frac{X_{\mathrm{ij}}}{({\sum_{j}X_{\mathrm{ij}}^{2})}^{1/2}}$$
6. Now each row of Y is treated as a sample, $s_{i}$, then all points are clustered into $k^{*}$ clusters using GMM. Spectrum uses the implementation of GMM from the ClusterR CRAN package.

**A flexible heuristic for finding *K* when spectral clustering.** A natural way to solve the problem of estimating *K* when spectral clustering is analysis of the eigendecomposition of the graph Laplacian. The classical eigengap method is effective for Gaussian clusters, however, its rule must be modified to detect non-Gaussian structures, thus limiting its applicability (see Section 3). We describe a new heuristic for finding *K* that can be used for Gaussian or non-Gaussian structures and as a complementary method to analyse genome-wide expression datasets. The method examines the multimodality of the eigenvectors of the graph Laplacian and looks for a point beyond which there is no more substantial decrease in multimodality.

Intuitively, the degree of multimodality defines how informative a given eigenvector is, and when we pass the optimal *K* moving along the sorted eigenvectors, $V=\left\{x_{1},\;x_{2},\;\ldots \;x_{N}\right\}$, we expect a large drop in useful information. Multimodality is quantified using the well-known dip test statistic (Hartigan and Hartigan, 1985). The method can work better (see Section 3) if the nearest-neighbour parameter of the kernel is tuned from P=1,…, 10. This is done by selecting the kernel that gives the maximum multimodality gap. Analysing the multimodality drop was inspired by the Xiang and Gong study (Xiang and Gong, 2008), in which the authors select the most informative eigenvectors using an expectation maximization (EM) technique, then use GMM and the BIC to choose *K*. An issue with this EM method is the instability of the results due to the random initialization of the algorithm and its local search. We now detail an alternative procedure for finding *K* and tuning the kernel based on decreases in eigenvector multimodality.

Let the set of dip test statistics be $Z=\left\{z_{1},z_{2},z_{3}\ldots ,z_{N}\right\}$, calculated from the eigenvectors, $V=\left\{x_{1},\;x_{2},\;\ldots \;x_{N}\right\}$. Note that larger values of $z_{i}$ correspond to greater eigenvector multimodality. To calculate the multimodality difference between consecutive values, we use $d_{i}=z_{i}-z_{i-1}$. Since we require two values to get $d_{i}$, the calculation must begin at i=2, which corresponds to the first pair of eigenvectors. Let the set of $d_{i}$ values calculated from Z be $D=\left\{d_{1},d_{2},d_{3}\ldots ,d_{\mathrm{maxK}-1}\right\},$ where maxK is the maximum value of K to be considered, the steps for this are as follows:

1. Find the optimal kernel, $A^{*}$. Each kernel is calculated using Equation (1) and the nearest-neighbour parameter P is tuned via a search from P=1,…, 10. To do this, calculate the ${P\mathrm{th}}^{}$ graph Laplacian [Equation (6)] from the ${P\mathrm{th}}^{}$ kernel. Obtain the eigenvectors of these, $V_{P}$. Calculate $Z_{P}$ from these eigenvectors, then $D_{P}$. Get $\min(D_{P})$ for each P, yielding a set $D_{\min}=\left\{d_{1},\;d_{2},\;\ldots \;d_{10}\right\}$. $A^{*}$ is the kernel that corresponds to $\min\left\{D_{\min}\right\}$.
2. Get the *k*NN graph of $A^{*}$, row normalize, then perform diffusion iterations [Equations(3)–(5)]. Optional.
3. Calculate the graph Laplacian, L [Equation (6)].
4. Perform eigendecomposition of L yielding the eigenvectors, $V=\left\{x_{1},\;x_{2},\;\ldots \;x_{N}\right\}$.
5. Calculate the dip test statistics Z for the eigenvectors in V, then calculate the differences of these, D.
6. Pass D into the algorithm described below for finding the last substantial drop in multimodality. Let $k^{*}$ be the optimal *K* found by this method.
7. Continue with steps 4–6 corresponding to the Ng spectral clustering method, with GMM to cluster the final eigenvector matrix, with *K* set to $k^{*}$.

One could select *K* using the maximum multimodality gap. However, we found that this simpler method is susceptible to local minima (see Section 3). This naturally led to making an algorithm to find the last substantial drop. For finding $k^{*}$ from set Z, we now describe this straightforward algorithm that reads along the elements of Z, to find a point where there is no more substantial decrease in multimodality.

**Finding last substantial multimodality gap.** This algorithm will search D, storing in memory the biggest difference in multimodality. Let that be $d_{\min}$. A more negative $d_{i}$ corresponds a bigger drop in the elements of Z. The method then examines if there are any more points ahead of this drop (up until ${<c}_{\max}$ points) that are $\leq \frac{d_{\min}}{f}$, where f is the minimum magnitude that the drop must be to replace $d_{\min}$. If a new $d_{\min}$ is found, this new difference is stored in memory. This process continues until no more substantial drops are found with the threshold $c_{\max}$ to stop the search. More specifically:

1. Skip the first element of D, $d_{1}$, as this corresponds to the drop from the first to second eigenvector, which is non-informative. Store in memory $d_{2}$, the greatest drop by default. Call this $d_{\min}$. Initialise a counter c=1 for keeping count of how many indices ahead we are of the stored $d_{\min}$.
2. Iterate from $d_{3}\ldots d_{\mathrm{maxK}-1}$ and with each iteration check if $\frac{d_{\min}}{f}>\;d_{i}$. If so, let $d_{i}$ be the new $d_{\min}$, otherwise continue. If $c>c_{\max}$, break the loop and accept the current stored $d_{\min}$ as the solution.
3. The optimal number of classes is $k^{*}=i-1$, where i is the index of D corresponding to the $d_{i}$ taken as $d_{\min}$ in step 2.

The parameters used in this study for the multimodality drop procedure were $c_{\max}=7$ and f=2, values we empirically selected based on our experience.

### 2.4 Generating simulated datasets for analysis

Gaussian cluster simulations were all performed using the CRAN clusterlab package (John *et al.*, 2018), following the standard operating procedure. In the case of non-Gaussian structures, found throughout the Supplementary Figures, either the CRAN mlbench or clusterSim packages were used to simulate the data, using the default settings.

### 2.5 Downloading and processing of real data for analysis

**TCGA datasets.** The seven multi-omic TCGA datasets (Agrawal *et al.*, 2014; Akbani *et al.*, 2015; Ceccarelli *et al.*, 2016; Ciriello *et al.*, 2015; Fishbein *et al.*, 2017; Network, 2013, 2014) were downloaded from the Broad Institute (http://gdac.broadinstitute.org/). Pre-normalized data were used for each platform (mRNA, miRNA and protein) and was log_2_ transformed to reduce the influence of extreme values. For every dataset each one was filtered in the same manner, using the coefficient of variation to select the top 50% most variable features. Code for data pre-processing is found in the following GitHub repository (https://github.com/crj32/spectrum_manuscript). The processed multi-omic data are in the Synapse repository syn18911550. RNA-seq datasets were taken from the same studies as the multi-omic data and filtering of features was done in the same manner. However, more patients were included in the RNA-seq analyses because we did not have to unify the patients between platforms. The RNA-seq data is included in the Synapse repository syn18911550. Code for performing log-rank tests in also in the GitHub as well as commands for running methods.

**Single-cell RNA-seq datasets.** The seven single-cell RNA-seq datasets (Baron *et al.*, 2016; Camp *et al.*, 2017; Darmanis *et al.*, 2015; Li *et al.*, 2017; Muraro *et al.*, 2016; Patel *et al.*, 2014; Pollen *et al.*, 2014) were obtained from the Hemberg lab website (https://hemberg-lab.github.io/scRNA.seq.datasets/). For each dataset, we used log_2_ normalized counts and selected the top 100 most variable genes for analysis. Code for data pre-processing is found in the manuscript’s GitHub (https://github.com/crj32/spectrum_manuscript). Additionally, we include the single cell RNA-seq data in the Synapse repository syn18911550. Code for calculating normalized mutual information (NMI) is in the GitHub as are commands for running methods.

## 3 Results

### 3.1 Spectrum provides fast effective clustering of single and multi-omic data

**Fig. 1.**
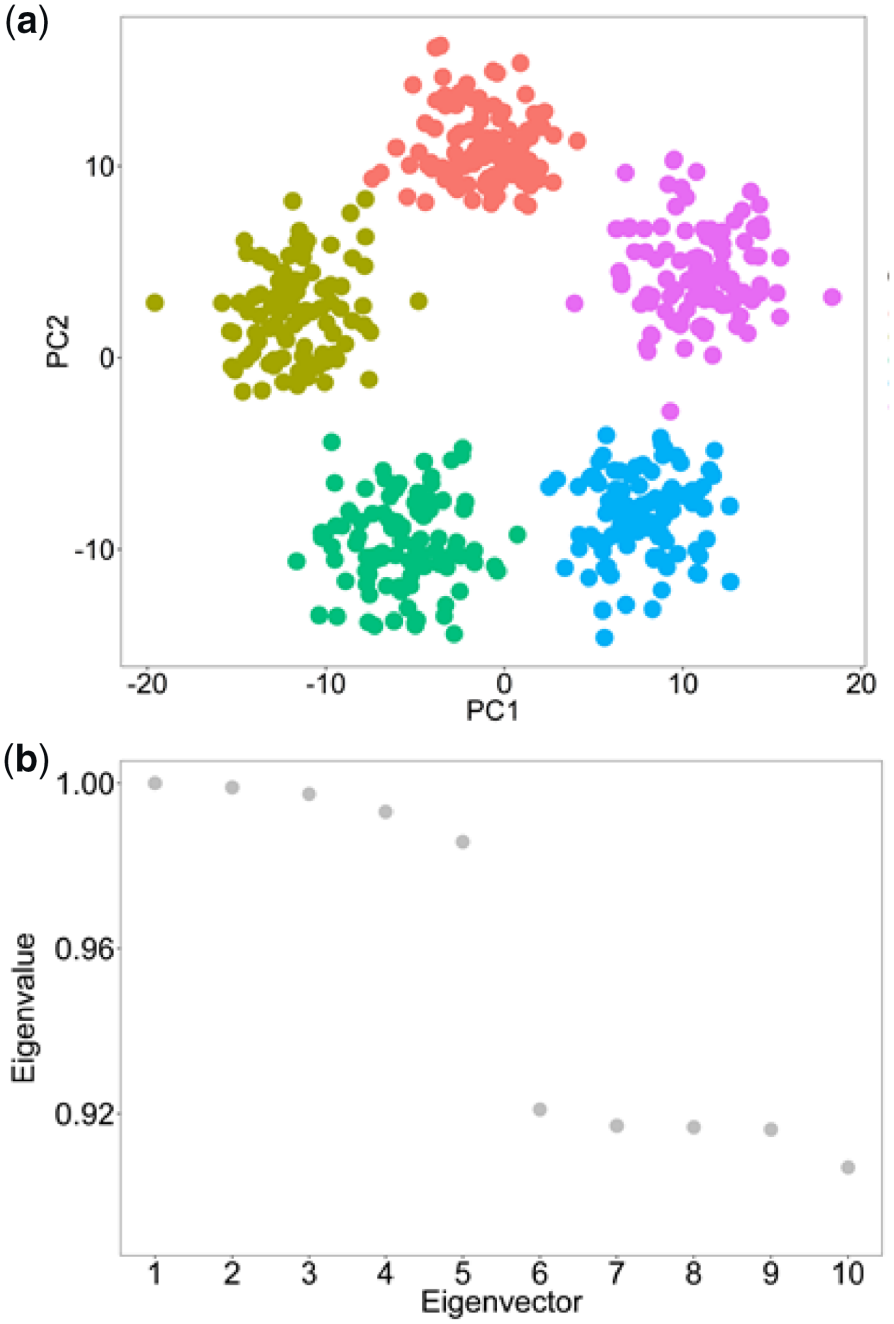
Spectrum clusters five simulated Gaussian clusters and finds the correct *K*. (**a**) PCA showing the five simulated Gaussian clusters. (**b**) The eigenvalues of the eigenvectors from the data’s graph Laplacian, the greatest eigengap is between the fifth and sixth eigenvectors, therefore correctly indicating *K *= 5

**Fig. 2.**
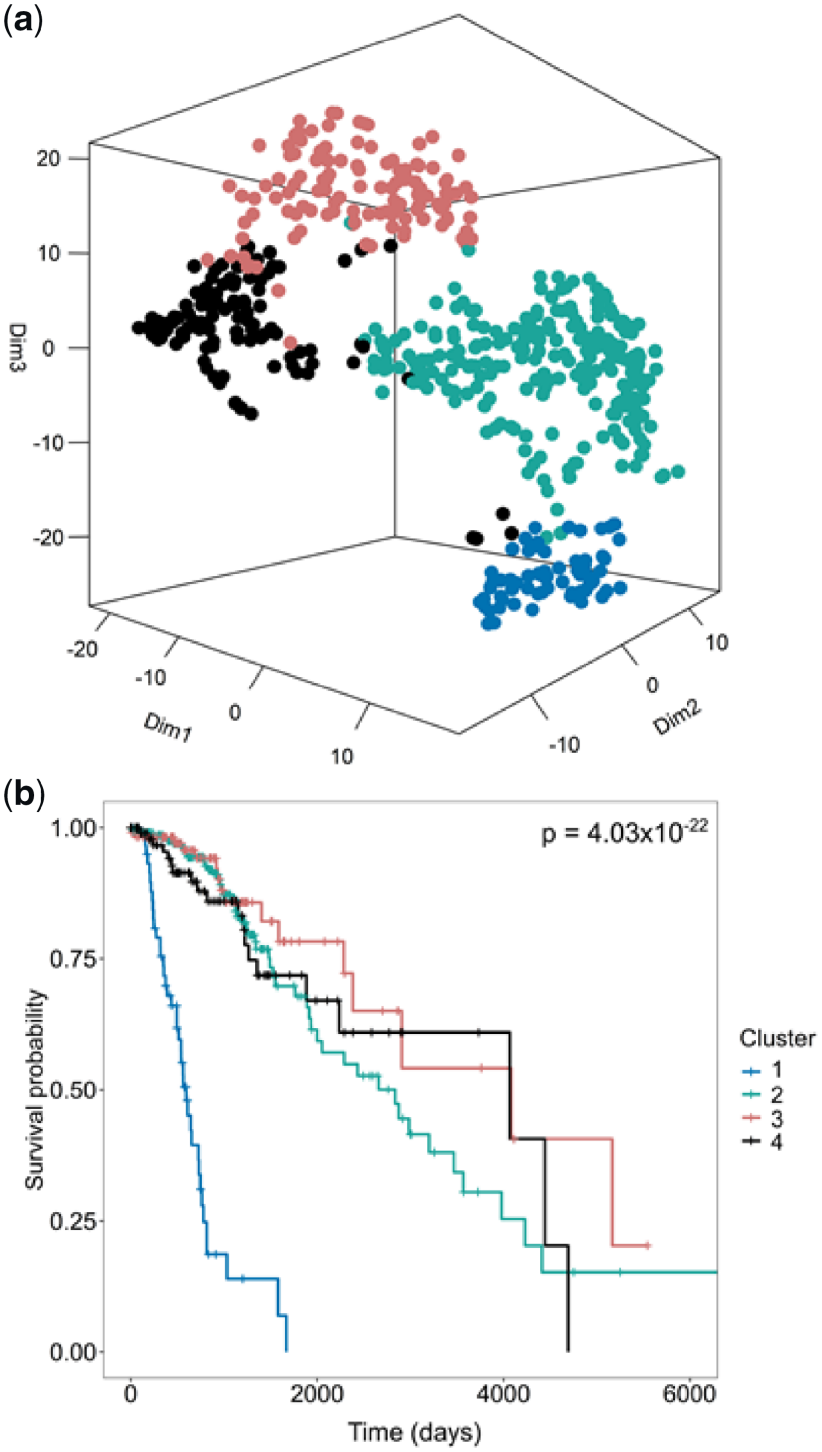
Spectrum clusters RNA-seq data to find cancer subtypes with different survival times. (**a**) t-SNE plot illustrating the four clusters Spectrum identified in a brain cancer RNA-seq dataset (Ceccarelli *et al.*, 2016). (**b**) Survival curve analysis results using the discovered clusters showing a *P*-value from a Cox proportional hazards regression model using a log-rank test to test the significance of the survival time differences between clusters

First, we tested Spectrum’s ability to identify the ground truth *K* on individual simulated Gaussian datasets (Fig. 1a and b and Supplementary Fig. S1). In each case, Spectrum correctly identified the optimal *K*. The method can also detect more complex non-Gaussian structures (Supplementary Fig. S2). To demonstrate the performance of Spectrum on real data from a single platform, we ran the algorithm on seven TCGA RNA-seq datasets (Agrawal *et al.*, 2014; Akbani *et al.*, 2015; Ceccarelli *et al.*, 2016; Ciriello *et al.*, 2015; Fishbein *et al.*, 2017; Network, 2013, 2014). We used log-rank tests to evaluate the significance of survival time differences between identified clusters. Comparison of Spectrum *P* values with those from CLEST, M3C, PINSplus, and SNF found that Spectrum performed better overall in finding clusters significantly related to patient survival (Supplementary Table S1). For comparing different methods, we took both a rank and *P*-value-based approach to assess performance, individual *P* values and rankings for each method on each RNA-seq dataset are included in Supplementary Table S1. The brain cancer RNA-seq dataset (Ceccarelli *et al.*, 2016) was used as an example to display the Spectrum single-omic clustering results using a *t*-distributed stochastic neighbour embedding (t-SNE) plot and the related survival curve was also shown (Fig. 2a and b). The clusters in the brain cancer dataset found with Spectrum were compared with those from SNF on t-SNE plots (Supplementary Fig. S3a and b). As well as obtaining a lower *P*-value, Spectrum yielded more compact clusters than SNF (measured by silhouette width). To give an initial indication of the relative computational resources required for a single platform analysis, algorithm runtime was investigated on a kidney cancer RNA-seq dataset (Network, 2013) with 240 points and 5000 features. This analysis was performed on a single core of an Intel Core i7-6560U CPU @ 2.20 GHz laptop computer with 16 GB of DDR3 RAM. Spectrum was the fastest method (1.13 s), closely followed by SNF (2.67 s). PINSplus was still fast (8.53 s), while M3C (123.91 s) and CLEST (283.34 s) were both slower.

To test the behaviour of Spectrum’s TPG integration method, we conducted a multi-omic data simulation where three Gaussian clusters were generated for each view, and each view had 300 points with 500 features with random noise added (Supplementary Fig. S4a). Individual platform clustering using Spectrum did not detect the optimal *K* (Supplementary Fig. S4b and c). However, using the TPG integration and diffusion method, Spectrum identified the optimal *K* for the combined dataset (Supplementary Fig. S4d). As expected, SNF also identified the optimal *K* on this simple test dataset (Supplementary Fig. S5). We proceeded to test Spectrum’s ability to detect clusters with significant differences in survival time on seven multi-omic TCGA datasets relative to other methods (Table 1). The analysis included mRNA, miRNA and protein data. Similar to our observations on a single platform, Spectrum performed very well, particularly on the larger datasets with greater potential for clinical significance, namely, the breast (*P* = 1.47E-07) and brain cancer (*P* = 3.76E-16) datasets.

**Table 1.** Spectrum multi-omic clustering performance relative to other algorithms

Dataset	*N*	Spectrum	PINSplus	iClusterPlus	SNF	CIMLR
Bladder	338	0.0042 (3)	0.31 (5)	0.0022 (2)	0.00022 (1)	0.0047 (4)
Brain	425	3.76E-16 (1)	0.0053 (4)	1.72E-07 (3)	4.17E-11 (2)	0.013 (5)
Breast	634	1.47E-07 (1)	2.85E-05 (4)	1.78E-05 (3)	0.94 (5)	2.04E-07 (2)
Kidney	240	0.91 (5)	0.038 (2)	0.24 (4)	0.045 (3)	0.0026 (1)
PCPG	80	0.043 (1)	0.18 (4)	0.093 (3)	0.09 (2)	0.54 (5)
Skin	338	0.0014 (1)	0.96 (5)	0.4 (3)	0.51 (4)	0.0029 (2)
Thyroid	219	0.049 (1)	0.09 (2)	0.67 (5)	0.18 (4)	0.17 (3)
P integrated		1.07E-22	1.04E-05	1.91E-10	2.22E-11	5.18E-11
Rank score		13	26	23	21	22

*Note*: *P* values are from a Cox proportional hazards regression model using a log-rank test to test the significance of the survival time differences between clusters. In brackets next to the *P* values are the ranks for each dataset. The first final row is the integrated *P*-value using Fisher’s method, the second is the sum of the ranks (lower is better). PCPG stands for Pheochromocytoma and Paraganglioma. For all datasets, the three data types used were mRNA, miRNA and protein.

We visually compared the multi-omic clusters found with Spectrum with those from SNF using uniform manifold approximation and projection (UMAP) plots for the first three multi-omic datasets (Supplementary Fig. S6a–c). Spectrum runs UMAP or t-SNE on the integrated similarity matrix as a new data visualization method for multi-omic data. The silhouette width was used as an additional scoring metric to the *P* values to investigate the quality of the clustering. On the brain and breast multi-omic datasets, Spectrum yielded higher silhouette widths than SNF, while for the bladder dataset the opposite was true. On the breast dataset SNF could be visually seen on the UMAP plot as missing a third cluster that Spectrum detected (Supplementary Fig. S6c). To investigate the consistency of the results from Spectrum and SNF, both methods were run on two parts of the randomly split brain cancer multi-omic dataset (Supplementary Fig. S7a and b). This dataset was chosen because it was the largest (*N* = 425), therefore likely to be stable in structure after splitting. Both Spectrum and SNF identified the same optimal *K* on each split, supporting their ability to perform consistently. These findings support the use of Spectrum as a complementary multi-omic spectral clustering tool to SNF and other methods.

Next, to gain an initial insight into relative multi-omic runtimes, we tested the algorithms on the kidney TCGA dataset (Network, 2013). Spectrum performed the fastest (2.5 s), followed by SNF (4.06 s), PINSplus (27.22 s), CIMLR (59.56 s) and iClusterPlus (305.35 s). A more extensive analysis of runtime was performed for the single and multi-omic algorithms using simulated data. This worked by increasing the number of points from 100 to 1000 in steps of 100, with each dataset containing 5000 features (Supplementary Fig. S8a and b). These analyses demonstrated the preferable runtimes of Spectrum relative to other methods. Spectrum’s good performance in finding clinically related clusters comes with a bonus of faster runtimes.

**Fig. 3.**
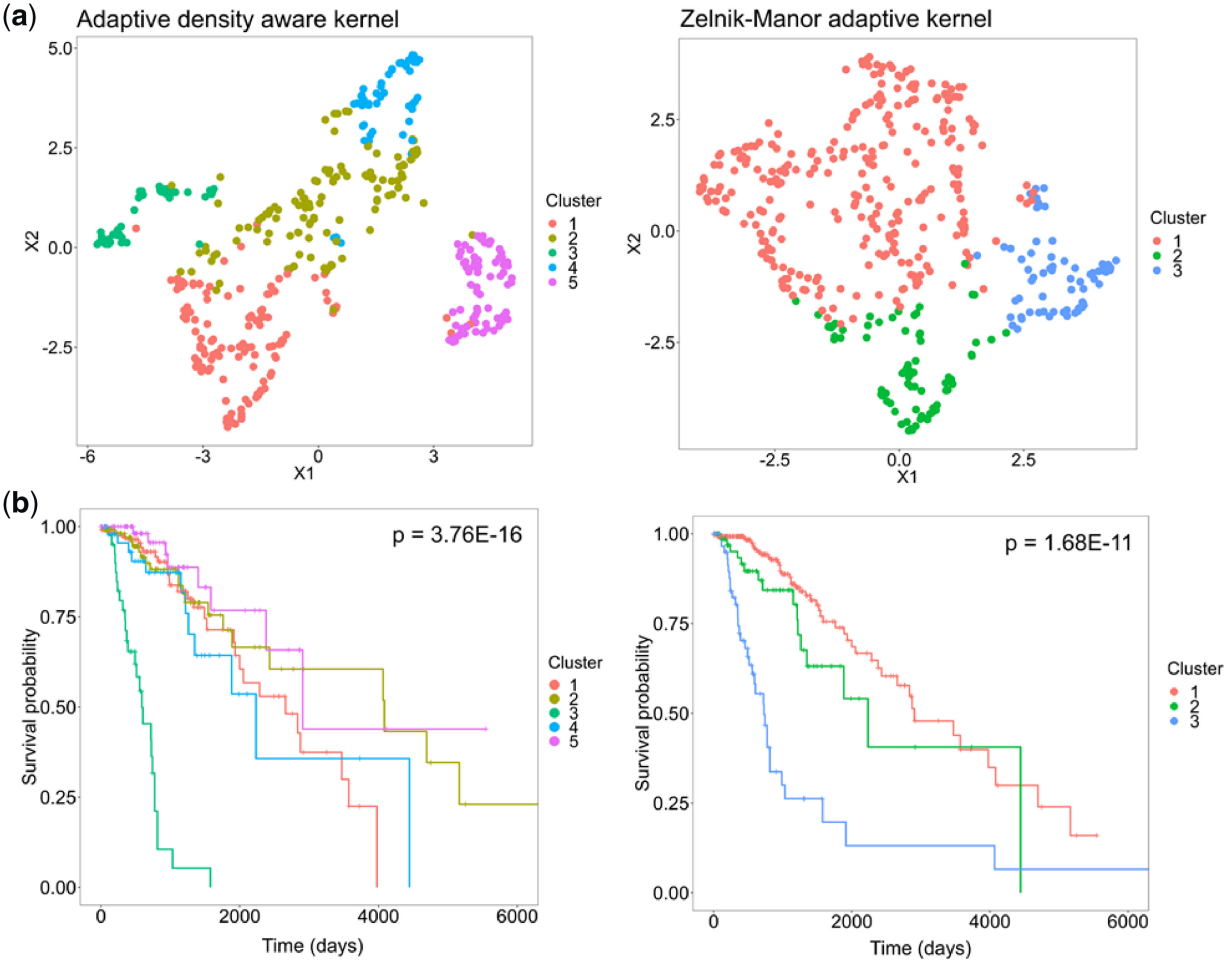
The adaptive density-aware kernel demonstrates an advantage in multi-omic analysis. On the right-hand side of the panel are the results for the Zelnik-Manor kernel, while the density-aware kernel results are shown on the left-hand side. (**a**) Spectrum clustering assignments from the brain cancer dataset (Ceccarelli *et al.*, 2016), UMAP was run on the integrated similarity matrices for mRNA, miRNA and protein data to generate the plots. (**b**) Survival curves with *P* values from a Cox proportional hazards regression model using a log-rank test to assess significance between clusters

We next demonstrated the advantage of Spectrum’s adaptive density-aware kernel by comparison with the classic Zelnik-Manor kernel, a non-density-aware kernel that adapts to local data scale only. First, Spectrum using either of the two kernels was run on a non-Gaussian synthetic dataset consisting of two worm-like structures. The clustering demonstrated that the density-aware kernel improved the classification (Supplementary Fig. S9a and b). Next, differences on TCGA multi-omic data were examined. Analysis of the brain cancer multi-omic dataset (Ceccarelli *et al.*, 2016) found the density-aware kernel detected two additional clusters in comparison with the Zelnik-Manor kernel (Fig. 3a). UMAP plots demonstrated the density-aware kernel results in visually more compact clusters than the Zelnik-Manor kernel. This was expected, as the density-aware kernel enhances connections in the graph where the points share common nearest neighbours. The survival *P* values produced by the different methods were shown on survival curves (Fig. 3b). Spectrum obtained a greater level of significance using the density-aware kernel (*P* = 3.76E-16) than using the Zelnik-Manor kernel (*P* = 1.68E-11). We expanded this comparison to include all seven TCGA multi-omic datasets to find that the density-aware kernel has a noticeable advantage over the Zelnik-Manor non density-aware kernel (Table 2). These findings demonstrate the potential for improvement gains by using a kernel that considers common nearest neighbours.

**Table 2.** Comparison of spectrum density-aware kernel versus the Zelnik-Manor self-tuning kernel in a multi-omic cluster analysis

Dataset	Data types	*N*	Spectrum density aware	Spectrum Zelnik-Manor
Bladder	mRNA, miRNA, protein	338	0.0042	0.0033
Brain	mRNA, miRNA, protein	425	3.76E-16	1.68E-11
Breast	mRNA, miRNA, protein	634	1.47E-07	3.56E-07
Kidney	mRNA, miRNA, protein	240	0.91	0.86
PCPG	mRNA, miRNA, protein	80	0.043	0.35
Skin	mRNA, miRNA, protein	338	0.0014	0.0058
Thyroid	mRNA, miRNA, protein	219	0.049	0.054
P integrated			1.07E-22	7.71E-17

*Note*: Values correspond to *P* values from a Cox proportional hazards regression model using a log-rank test to test the significance of the survival time differences between clusters. The final row is the integrated *P*-value using Fisher’s method. PCPG stands for Pheochromocytoma and Paraganglioma.

### 3.2 Spectrum performs well at identifying cell types in single-cell RNA-seq data

We examined Spectrum’s performance on simulated datasets that resemble single-cell RNA-seq, as they were made to consist of many Gaussian blobs that can overlap. Spectrum identified the correct *K* for both the *K* = 10 simulated dataset and the *K* = 20 dataset (Supplementary Fig. S10a–d). Next, we tested Spectrum’s performance relative to other methods on seven real single-cell RNA-seq datasets (Baron *et al.*, 2016; Camp *et al.*, 2017; Darmanis *et al.*, 2015; Li *et al.*, 2017; Muraro *et al.*, 2016; Patel *et al.*, 2014; Pollen *et al.*, 2014) by comparing the assigned clusters with the provided cell type labels using NMI (Supplementary Table S2). Spectrum had the highest summed NMI (NMI = 5.89), closely followed by Seurat (NMI = 5.74), MUDAN (NMI = 5.71), SC3 (NMI = 5.49) and SIMLR (NMI = 5.01). Spectrum’s summed NMI was favourably weighted by its performance on the Pollen dataset (NMI = 0.95). Using a rank-based score to eliminate this advantage, Spectrum still came joint first with SC3 (Supplementary Table S2). A similarity matrix for the Pollen data results was shown and t-SNE plots showing the Spectrum clustering assignments were produced (Supplementary Figs S11 and S12). Overall, Spectrum, Seurat, SC3 and MUDAN performed similarly in these comparisons, however, SIMLR did not perform as well (Supplementary Table S2).

Notably, in the comparative analysis shown in Supplementary Table S2, since the Baron and Muraro datasets had higher numbers of points, to reduce runtime Spectrum was run using the FASP method (with 900 centroids). Even with the FASP data compression for these two datasets, Spectrum yielded the highest NMI relative to the other methods. Comparing Spectrum runtime on the Baron dataset (*N* = 8569) yielded 1.95 h without FASP versus 14.23 s with FASP. Analyses were performed on a single core of an Intel Core i7-6560U CPU @ 2.20 GHz laptop computer with 16 GB of DDR3 RAM. On the Muraro dataset (*N* = 2126), without FASP took 1.97 min and with took 11.83 s. Since the complexity of spectral clustering is cubic,$\;O\left(N^{3}\right)$ and the complexity of *k* means is linear, $O\left(\mathrm{KNT}\right)$, where T is the number of *k* means iterations, using *k* means as a precursor to compress the data (FASP) is computationally advantageous on larger datasets.

To gain an initial insight into relative runtimes of all methods (without using Spectrum’s FASP implementation), methods were run on the Camp dataset (777 points). This analysis found MUDAN performed the fastest (0.23 s), followed by Seurat (2.45 s), Spectrum (12.64 s), SC3 (183.66 s) and SIMLR (264.31 s). A more detailed runtime analysis was performed for all algorithms on simulated datasets with 500 to 4000 points in steps of 500 with 1000 features (Supplementary Fig. S13). Spectrum was in the middle in terms of speed, usually performing faster than the SC3 algorithm. However, SC3 adjusted its own parameters to work faster at higher numbers of points making it of comparable speed to Spectrum. Spectrum was slower than MUDAN and Seurat, but much faster than SIMLR. Overall, these data demonstrate Spectrum is well suited to clustering small to large single cell RNA-seq datasets, with FASP required for the later.

### 3.3 A fast new heuristic for finding *K* when performing spectral clustering

Since the eigengap method does not automatically recognize both Gaussian and non-Gaussian structures (Supplementary Figs S1 and S2), we developed a complementary method which can. The method involves examining the multimodality of the eigenvectors of the data’s graph Laplacian, so we call it ‘the multimodality gap’. To demonstrate this method, five Gaussian blobs were generated (Supplementary Fig. S14a) and the multimodality of the data’s graph’s eigenvectors were also displayed (Supplementary Fig. S14b). The dip-test statistic (Hartigan and Hartigan, 1985) (*Z*) which measures multimodality demonstrated a large gap between eigenvectors five and six. Therefore, using this method it was correctly concluded that *K* = 5. Each individual eigenvector was plotted out (Supplementary Fig. S14c) to demonstrate the changing distribution of the eigenvectors. As observed in the analysis of the set of *Z* values, there was a transition from a multimodal distribution at eigenvector five to a unimodal distribution at eigenvector six, supporting *K *= 5. To further demonstrate the method, several simulations were run and the method successfully clustered both complex non-Gaussian (Supplementary Fig. S15a–d) and Gaussian clusters (Supplementary Fig. S16a–d). However, since the simple method of looking for the greatest gap in the set of *Z* values can get stuck in local minima (Supplementary Fig. S16d), the method was further enhanced by adding an algorithm to search for the last substantial gap.

We found the multimodality gap requires kernel tuning to perform well on certain datasets. This was evident in non-Gaussian data simulations, as with kernel tuning there is a perfect clustering result for the spirals test data (Supplementary Fig. S17a), while without kernel tuning the method fails to cluster correctly (Supplementary Fig. S17b). Kernel tuning is performed by simply changing the P parameter of the self-tuning kernel and for each kernel finding the maximum multimodality gap between any pair of eigenvectors. The kernel that yields the greatest gap is the optimal kernel, where the most negative *D* value corresponds to that kernel with the maximum gap (Supplementary Fig. S17c). We examined the performance of the multimodality gap across the seven TCGA multi-omic datasets to demonstrate its applicability as an alternative method to the eigengap. This analysis found the multimodality gap can provide different *P* values compared with the eigengap (Supplementary Table S3). Preferable methods will vary according to the data. For example, the multimodality gap (*P* = 0.0019) has a lower *P*-value than the eigengap (*P* = 0.91) on the kidney cancer data (Network, 2013). Including a second method to automatically decide *K* gives the user power to find the best approach for their data and presents a solution to an open problem in spectral clustering.

## 4 Discussion

Spectrum provides density-aware spectral clustering for complex omic data. Spectrum adapts to each new dataset by using each point’s *k*-nearest neighbours and their distances, instead of parameters that require tuning with each new dataset (Ng *et al.*, 2002; Zhang *et al.*, 2011), when performing kernel calculations. This enables the method to work quickly and yield good results. Spectrum was the fastest method in the single-omic and multi-omic TCGA data analysis. Our data also demonstrate good performance with Spectrum as lower *P* values and higher NMI values are often obtained on real data. This is partially due to the density-aware kernel that considers more local statistical properties of the data other than scale (Zelnik-Manor and Perona, 2005). Increasing the similarity between samples that share more common nearest neighbours enhances intra-cluster similarity. This produces more compact clusters and reinforces the underlying structure.

SNF was the first multi-view spectral clustering method to be developed for multi-omic data (Wang *et al.*, 2014). Spectrum is in the same family of algorithms; however, it does not include methods developed in the SNF study or advance upon them. Spectrum has several differences: (i) A different kernel that adapts to local density by strengthening local connections between points that share common nearest neighbours. (ii) A different data integration method that uses a TPG integration and diffusion technique. (iii) An alternative method for finding *K* that analyses eigenvector distributions. (iv) Use of GMM instead of *k*-means to cluster the graph’s eigenvectors. GMM can detect clusters with different variance and is preferred for spectral clustering (Zelnik-Manor and Perona, 2005). (v) Spectrum performs *k*NN graph diffusion on a single-view whereas SNF does not. This is because SNF only performs cross-diffusion between two or more different *k*NN graphs (from different data views). *k*NN graph diffusion is valuable, as has been demonstrated to reduce noise (Shu and Latecki, 2016). Spectrum is a novel and complementary tool.

Spectral clustering represents one of the most popular and promising techniques to integrate multi-omic data, partly because of the many well-established multi-view data integration methods developed in computer science (Kumar *et al.*, 2011; Rappoport and Shamir, 2018; Shu and Latecki, 2016; Wang *et al.*, 2014). However, there are other interesting types of integrative methods not tested in our study, including MANCIE 

(Zang *et al.*, 2016). MANCIE uses a correlation-based method that allows one data view to modify data in a second view by taking the first principal component or a weighted mean of the data. Another method, NBS (Hofree *et al.*, 2013) projects binary somatic mutation data from cancer tumours onto public gene–gene interaction networks. Network propagation is applied to spread the influence of each mutation profile over its neighbourhood network. The result is a matrix of continuous values for each gene that reflect the network proximity of the gene to mutated genes in that patient. This matrix can then be used for clustering. There have also been efforts elsewhere to combine somatic mutation data with pathway information to subtype cancer patients (Wang *et al.*, 2018).

Spectrum could be used to integrate somatic mutation data from tumours if the data were first made continuous, for example, as in the NBS method. To accept binary data directly, the kernel would have to be modified. We leave this for future work. The next step for Spectrum is to allow for missing data when performing integrating multi-omic data. This was recently proposed in the NEMO spectral clustering algorithm that uses mean imputation at the similarity matrix level (Rappoport and Shamir, 2018).

The multimodality gap heuristic for finding *K* increases the flexibility of Spectrum. This is because it can recognize both complex shapes and Gaussian clusters. There are few good solutions to this problem, none of which are implemented in a publicly available R library. The Zelnik-Manor self-tuning algorithm (Zelnik-Manor and Perona, 2005) involves a gradient descent technique that is complex to code and time consuming to execute. In contrast, the multimodality gap is relatively straightforward to implement, effective, and can be used to tune the kernel. Non-Gaussian clusters may occur in flow cytometry data (Zare *et al.*, 2010) and in image analysis (Xiang and Gong, 2008). Overall, Spectrum is a fast, sophisticated and efficient clustering method and is well suited to clustering a range of data.

## Supplementary Material

btz704_Supplementary_Data
