# Supplementary material for: Spectrum: fast density-aware spectral clustering for single and multi-omic data
Source: Bioinformatics. 2019 Sep 10;36(4):1159–66. doi: 10.1093/bioinformatics/btz704 (PMC7703791; doi:10.1093/bioinformatics/btz704)
Supplement: btz704_Supplementary_Data [file btz704_supplementary_data.pdf]

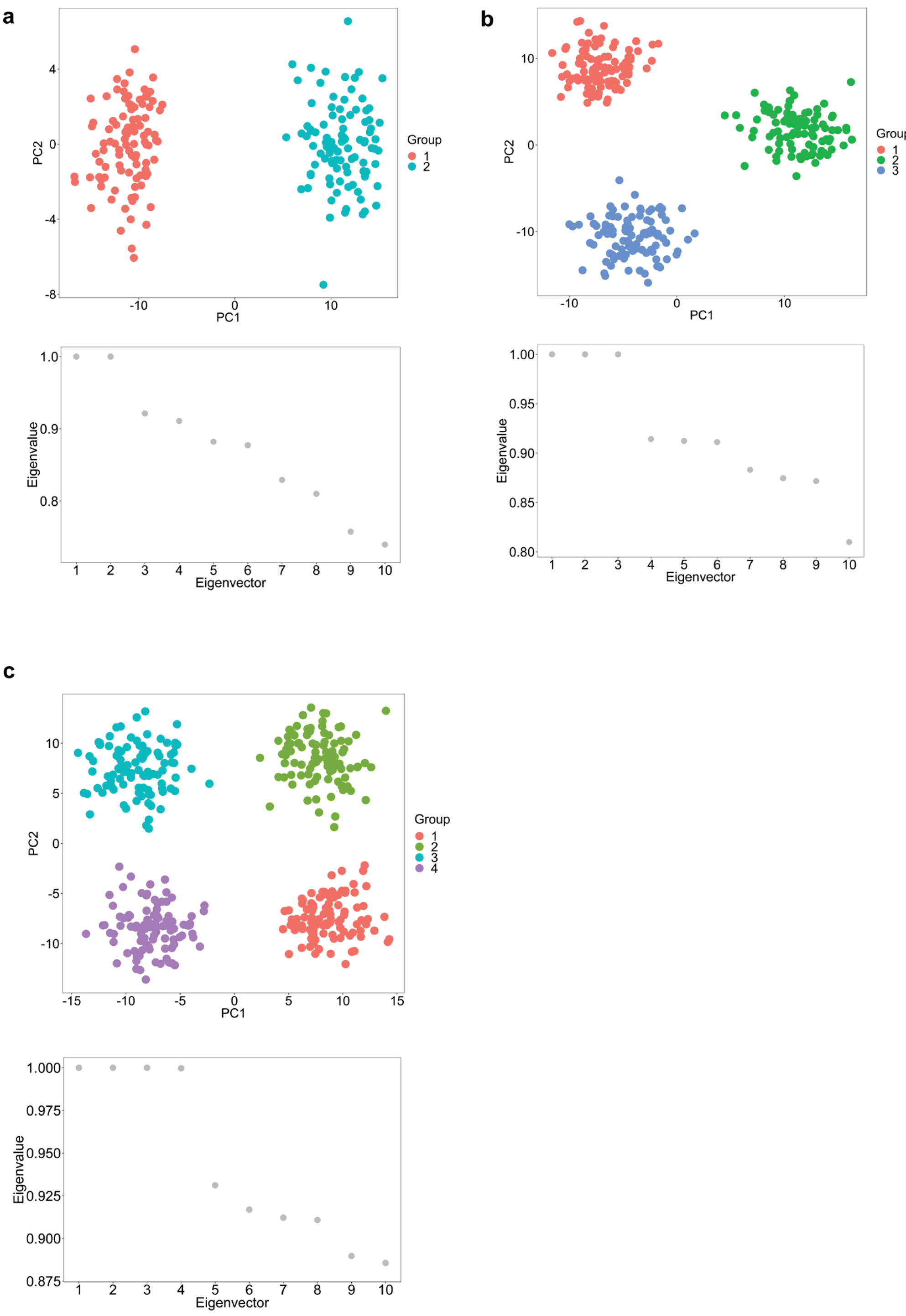

Supplementary Fig. S1. Spectrum performs well at identifying synthetic Gaussian clusters using the eigengap method. In each case a Principal Component Analysis (PCA) of the synthetic data is shown alongside the eigenvalues of the eigenvectors of the data's graph Laplacian. (A) Results from synthetic Gaussian data analysis with K=2. (B) Analysis for K=3. (C) Analysis for K=4. In all cases the maximum eigengap method identified the correct number of clusters. This simulation represents clustering data from a single omic platform.

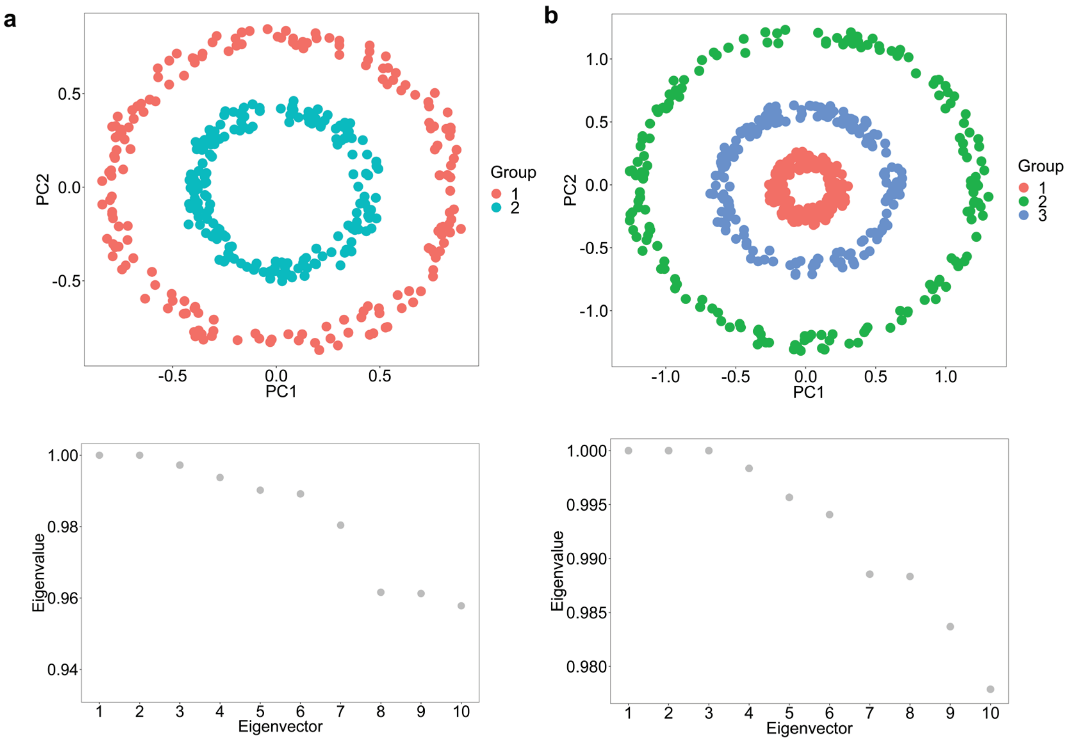

Supplementary Fig. S2. Spectrum performs well at identifying non-Gaussian clusters using the eigengap method with a modified decision rule. In each case a PCA of the synthetic data is shown alongside the eigenvalues of the eigenvectors of the data's graph Laplacian. (A) Results from synthetic K=2 concentric circles data analysis. (B) Analysis from K=3 concentric circles data analysis. In both cases the first non-zero eigengap method identified the correct number of clusters.

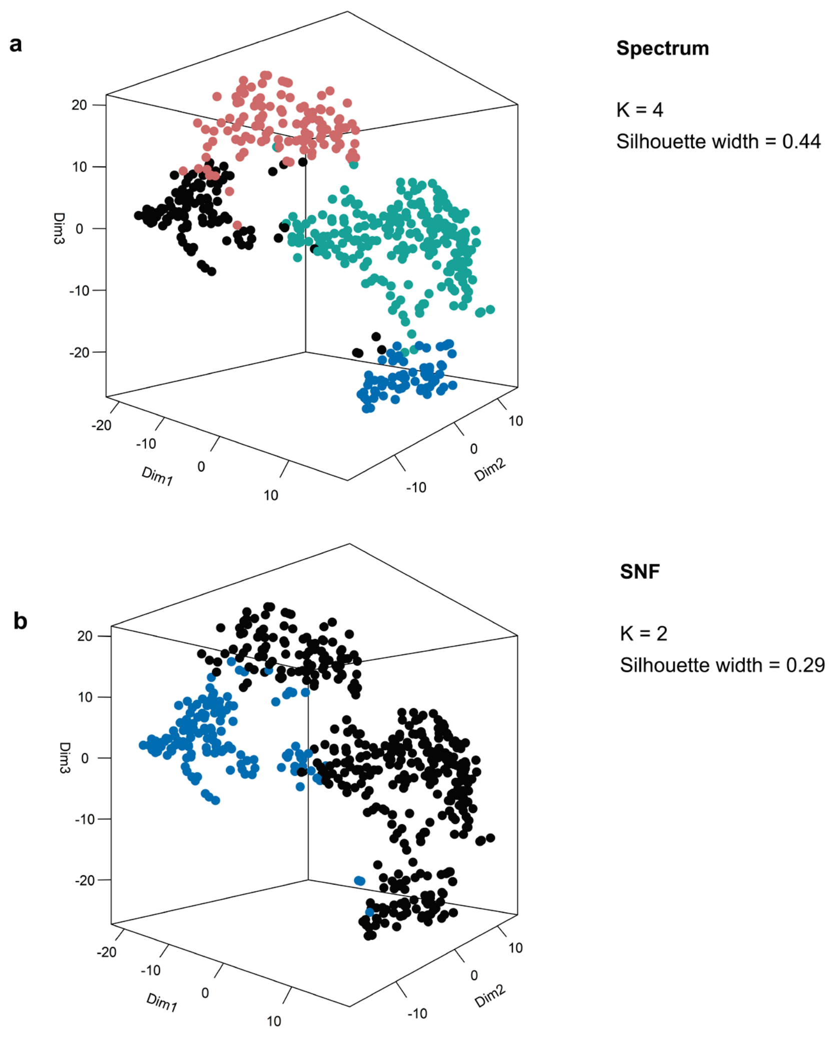

Supplementary Fig. S3. Comparison of Spectrum and SNF clustering results on a single-omic test dataset. (A) t-SNE plot illustrating the four clusters Spectrum identified in a brain cancer RNA-seq dataset. (B) t-SNE illustrating the two clusters SNF identified in the same data. Mean silhouette widths are given for both sets of clustering, higher mean silhouette width refers to preferable clustering results.

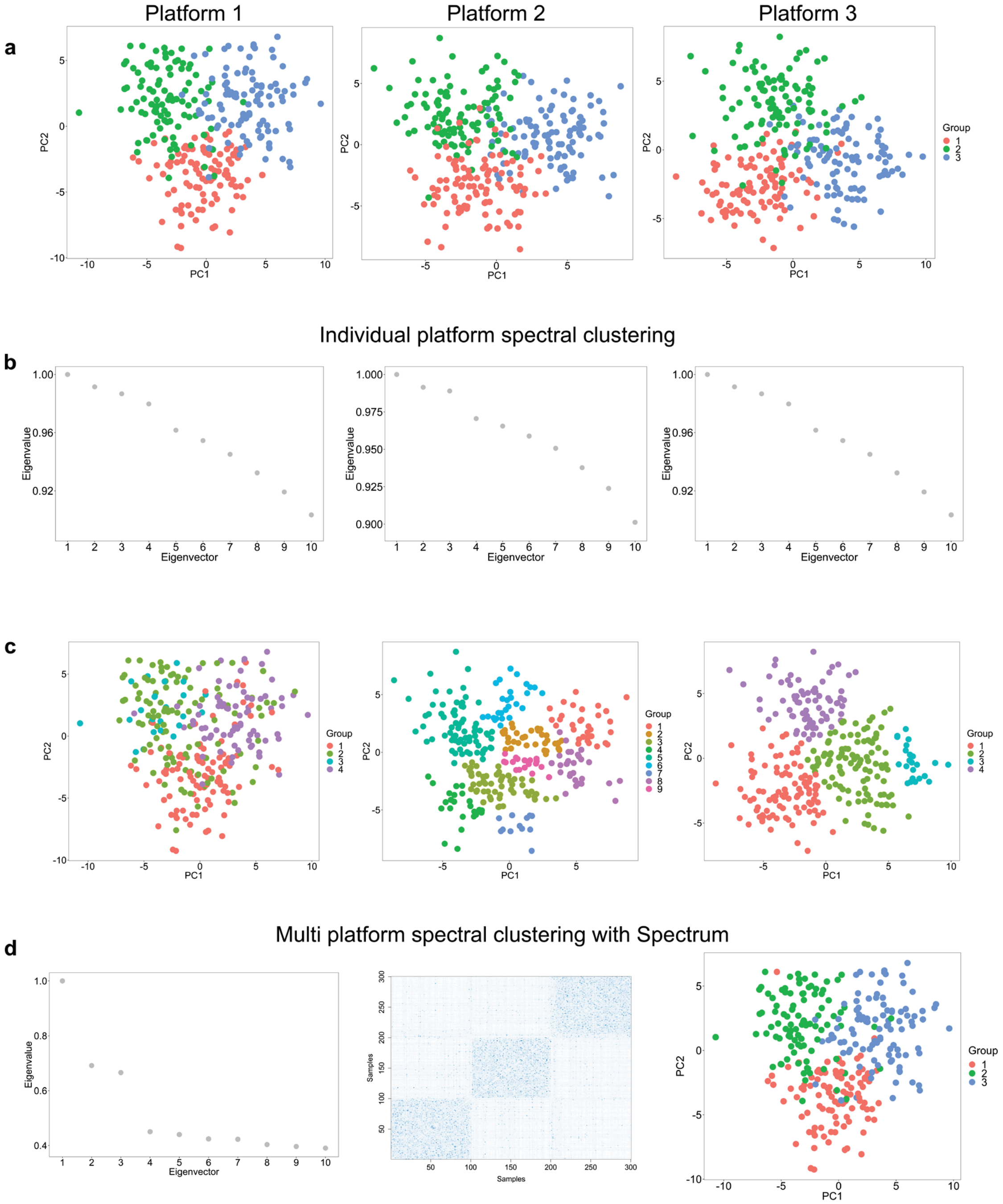

Supplementary Fig. S4. Spectrum performs well at reducing noise when clustering data generated in a multi-omic data simulation. (A) Three Gaussian clusters are simulated, each sample belongs to the same cluster in each simulation, but a degree of variability has been added. (B) Individual platform clustering could not detect the optimal K in every case due to noise. (C) Cluster assignments shown in PCAs using individual platforms with no data integration. (D) Spectrum data integration method results in finding K=3 on the combined data. In this simulation, three Gaussian clusters were generated for each view, and each view had 300 points with 500 features with random noise added to each point.

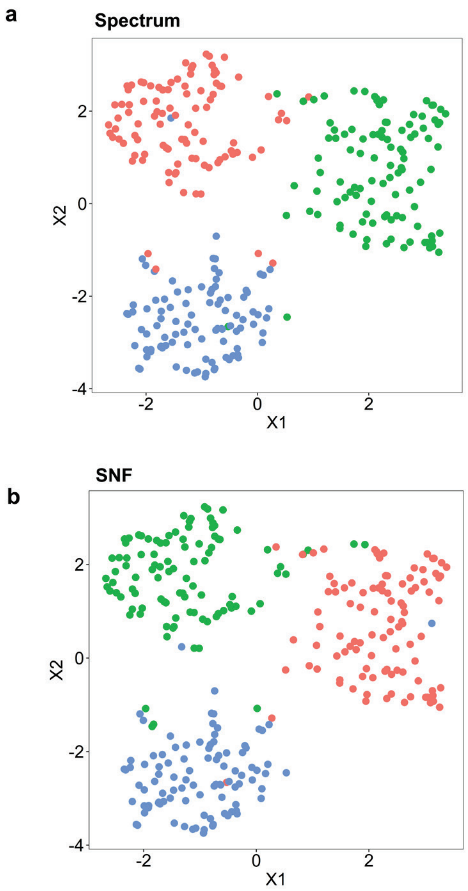

Supplementary Fig. S5. Comparison of Spectrum and SNF clustering results on the multi-omic data simulated data. (A) Clustering assignments from Spectrum displayed on a UMAP plot of the integrated similarity matrix. (B) Same as A but using SNF.

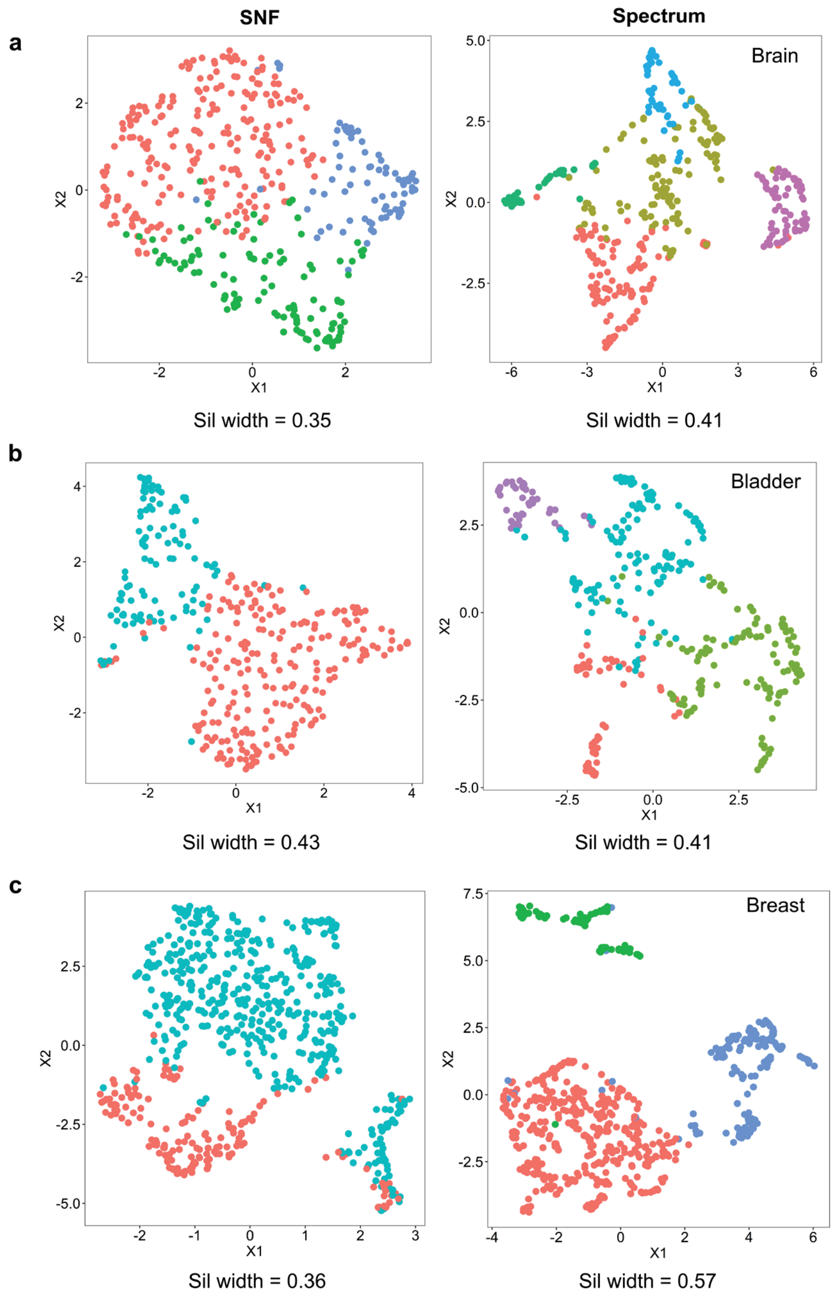

Supplementary Fig. S6. Comparison of Spectrum and SNF clustering results on three multi-omic test datasets. In each case UMAP was run on the integrated similarity matrix generated by either Spectrum or SNF. (A) UMAP plot illustrating the clusters Spectrum and SNF identified in a brain cancer multi-omic dataset(Ceccarelli, et al., 2016). (B) UMAP plot illustrating the clusters Spectrum and SNF identified in a bladder cancer multi-omic dataset(Network, 2014). (C) UMAP plot illustrating the clusters Spectrum and SNF identified in a breast cancer multi-omic dataset(Ciriello, et al., 2015). Mean silhouette widths are given for both sets of clustering, higher mean silhouette widths refer to preferable clustering results.

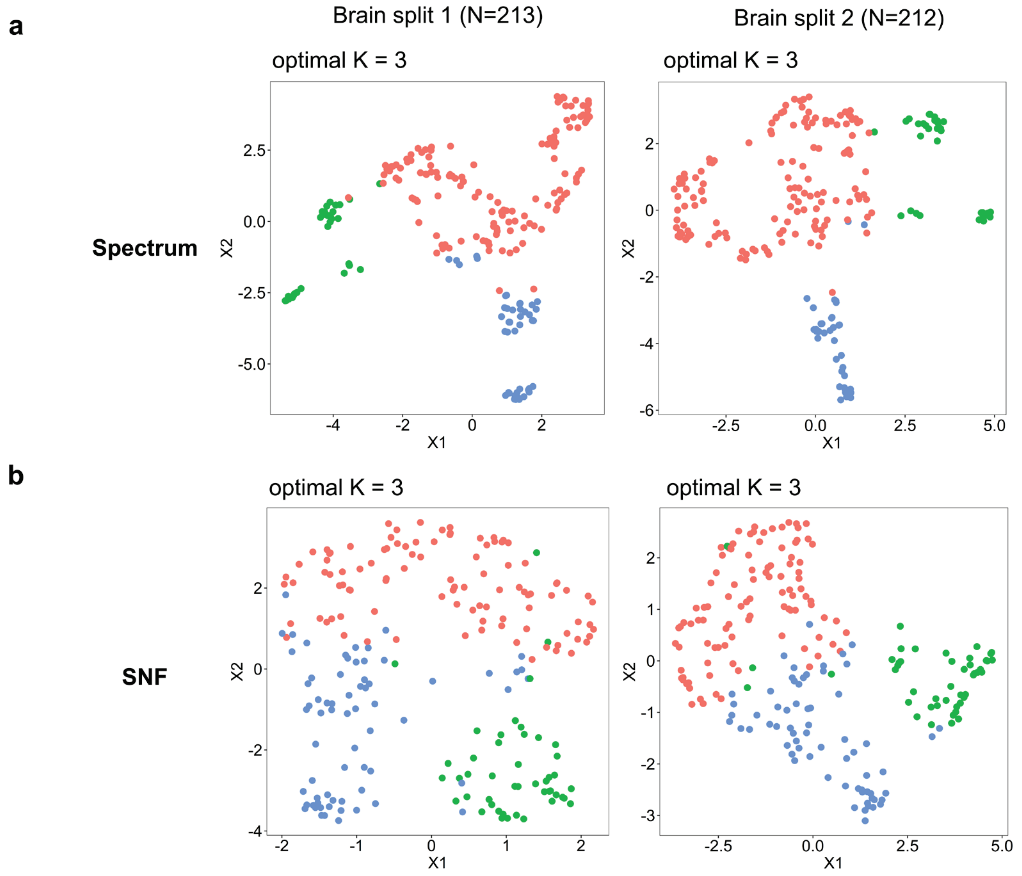

Supplementary Fig. S7. Comparison of the consistency of Spectrum and SNF clustering results. The brain cancer multi-omic data was randomly divided into two parts, the number of patients in each part is indicated by N. (A) UMAP plots showing Spectrum clustering assignments for each of the two random splits of the brain cancer dataset, in each case the same optimal K was identified. (B) Same, but for SNF clustering assignments.

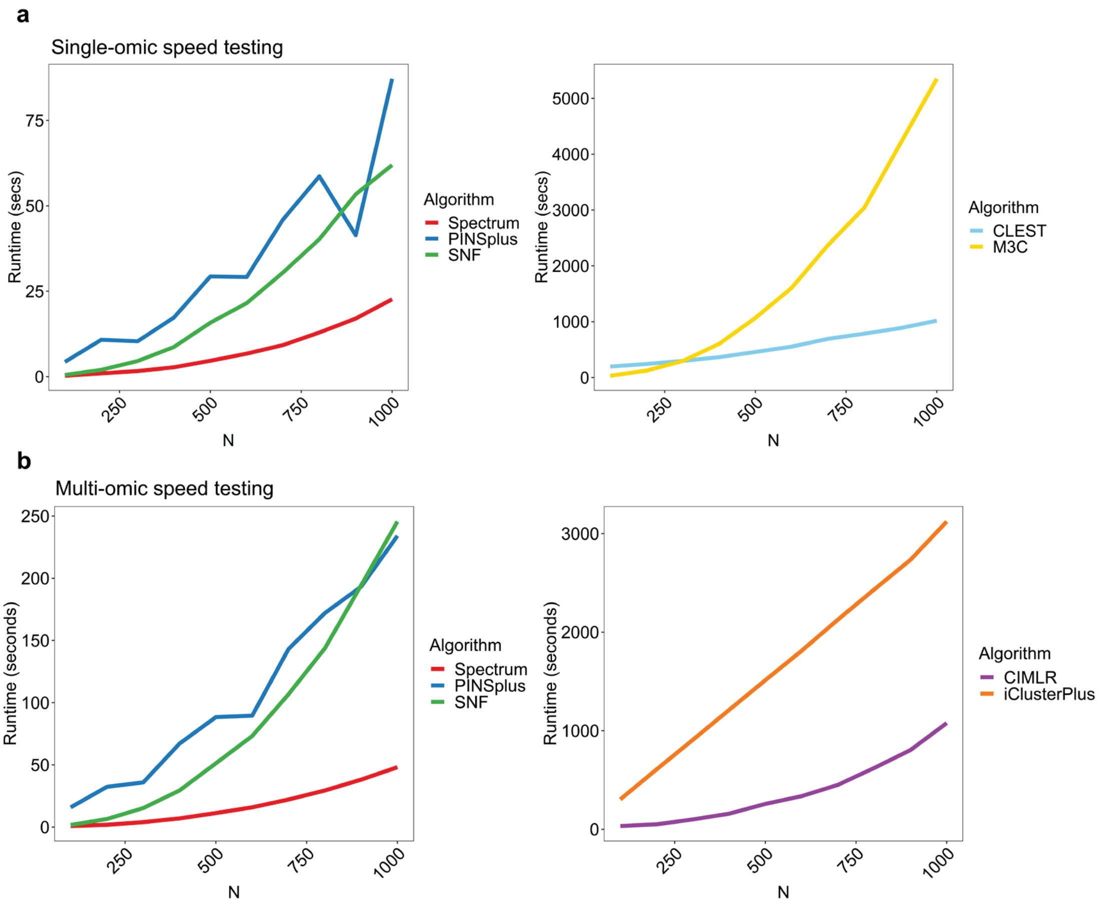

Supplementary Fig. S8. Spectrum works quickly on single and multi-omic TCGA data. (A) Runtime analysis for single-omic clustering algorithms. (B) Runtime analysis for multi-omic clustering algorithms. In each case the slower algorithms are shown on the right-hand side of the panel, while on the left-hand side faster ones.

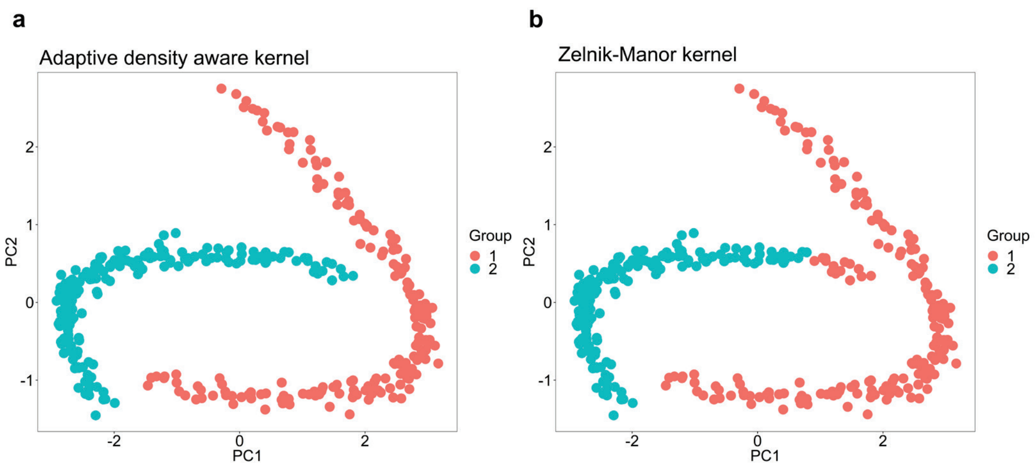

Supplementary Fig. S9. The locally adaptive density-aware kernel outperforms the Zelnik Manor kernel on a non-Gaussian dataset. (A) Spectrum results with the adaptive density-aware kernel. (B) Spectrum results with the Zelnik Manor non density-aware kernel.

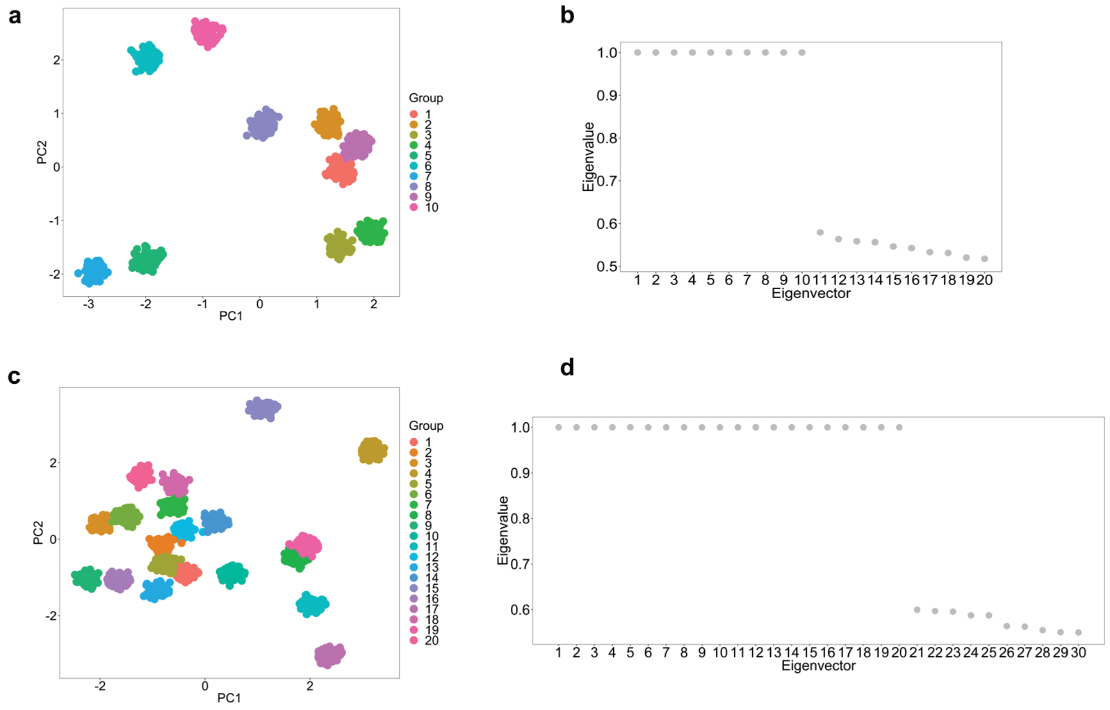

Supplementary Fig. S10. Spectrum performs well on simulated Gaussian data resembling single-cell RNA-seq. (A) PCA of the K=10 synthetic dataset. (B) Eigenvalues of the eigenvectors of the data's graph Laplacian, a maximum drop is located between 10 and 11 correctly designating this as the optimal K decision by the algorithm. (C) PCA of the K=20 synthetic dataset. (D) Eigengap method confirms K=20.

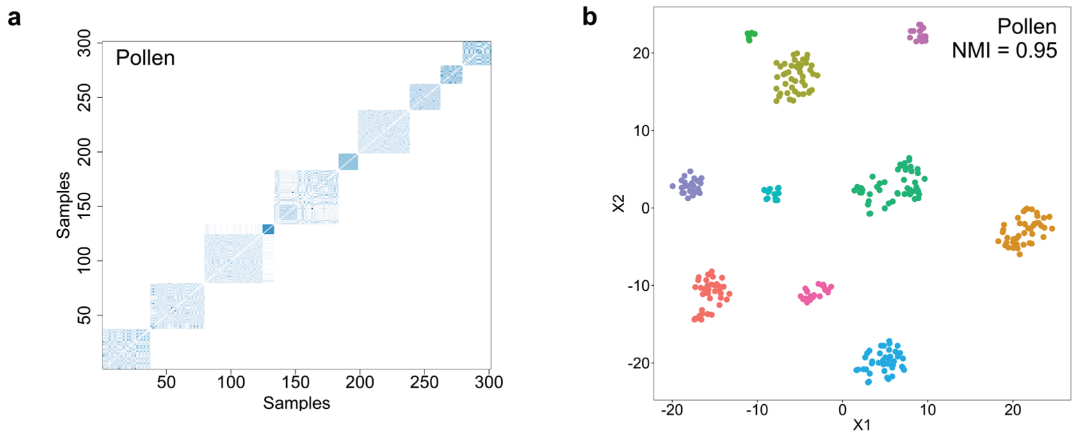

Supplementary Fig. S11. Spectrum identifies cell types in a single cell RNA-seq cluster analysis. (A) Heatmap of similarity matrix from Spectrum for the Pollen dataset. (B) t-SNE of the Pollen data overlaid with Spectrum clustering assignments. Normalised mutual information (NMI) between the cell labels and the detected clusters is shown.

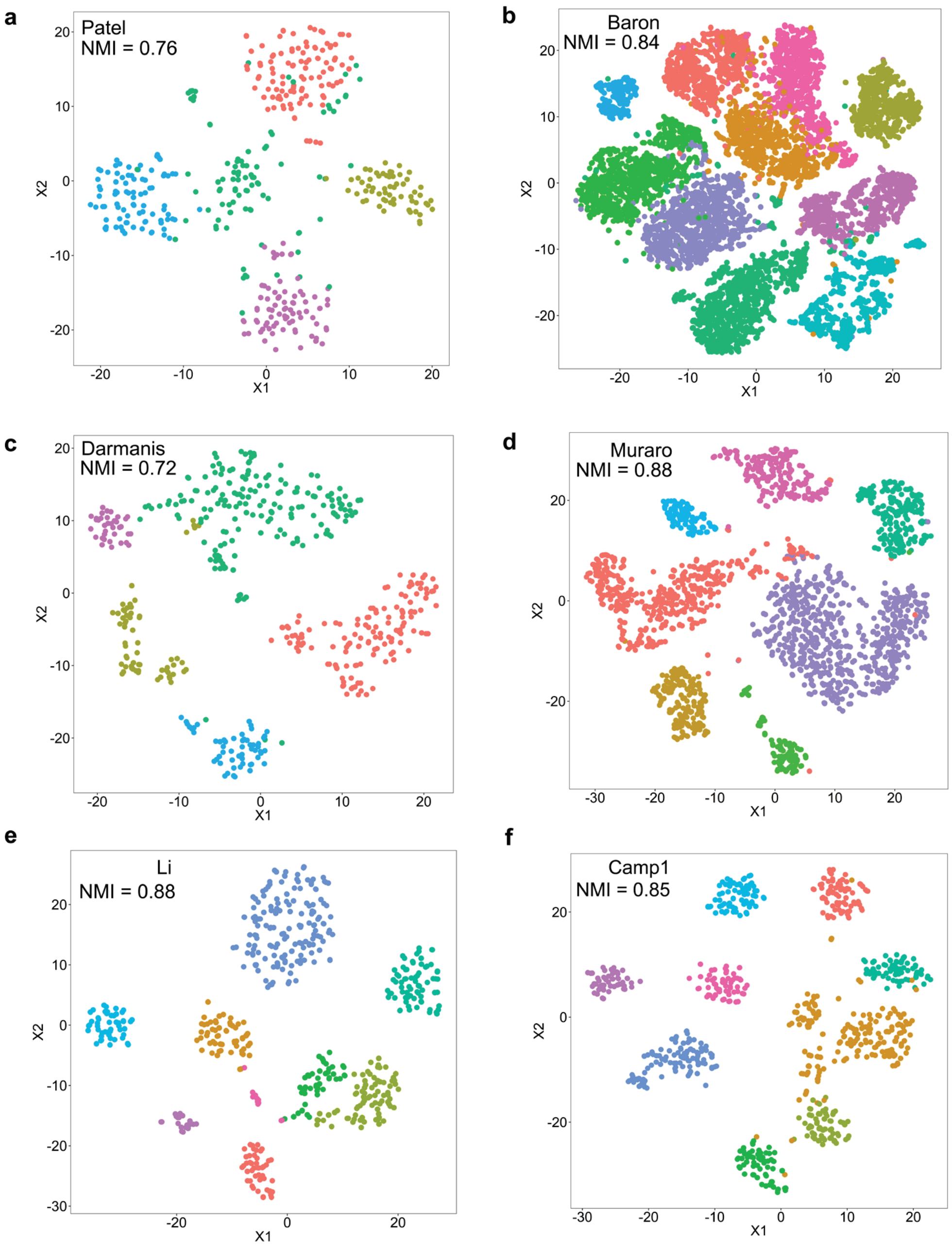

Supplementary Fig. S12. Spectrum single cell RNA-seq clustering results. (A) Results from t-SNE analysis of the Patel data overlaid with Spectrum clustering assignments. NMI between the cell labels and the detected clusters is shown. (B) Same as A, but for the Baron data. (C) Same as A, but for the Darmanis data. (D) Same as A, but for the Muraro data. (E) Same as A, but for the Li data. (F) Same as A, but for the Camp1 data.

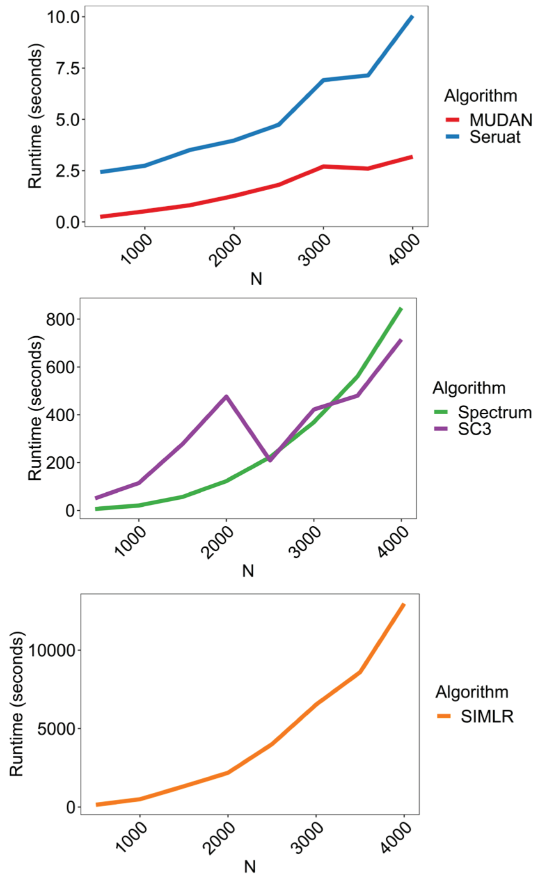

Supplementary Fig. S13. Runtime analysis for single-cell RNA-seq clustering methods. These analyses were run on a single core of an Intel Core i7-6560U CPU @ 2.20GHz laptop computer with 16GB of DDR3 RAM. Simulated datasets had 1000 features and varying numbers of points (N).

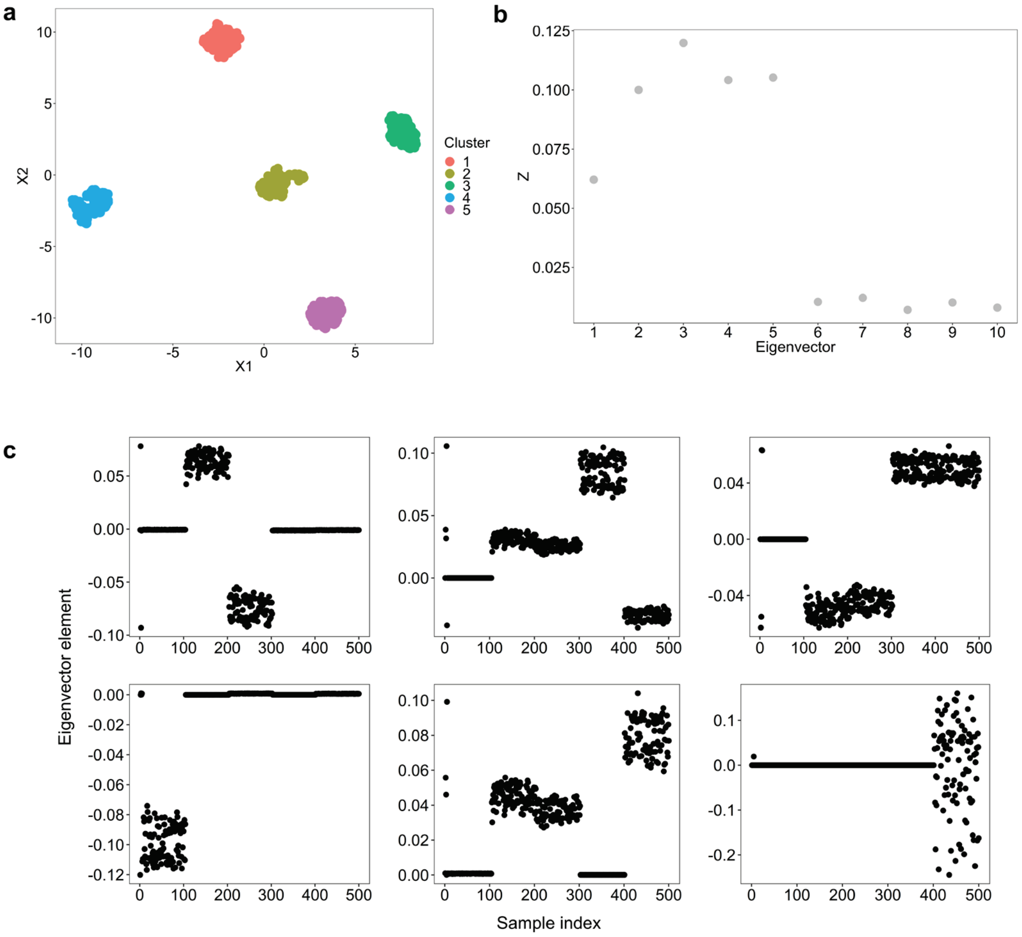

Supplementary Fig. S14. Demonstration of the concept behind the multimodality gap method. Z refers to the dip test statistic z_i for the ith eigenvector, where a larger value indicates stronger multimodality. (A) Five Gaussian clusters were simulated for testing of the method. (B) The eigenvectors of the data's graph Laplacian were tested using the dip test for multimodality, and a large drop in Z between the fifth and sixth eigenvectors was observed reflecting an optimal K of five. (C) Elements of each eigenvector were plotted out to observe the change from the fifth eigenvector of a more multimodal distribution to the sixth eigenvector of a more unimodal distribution.

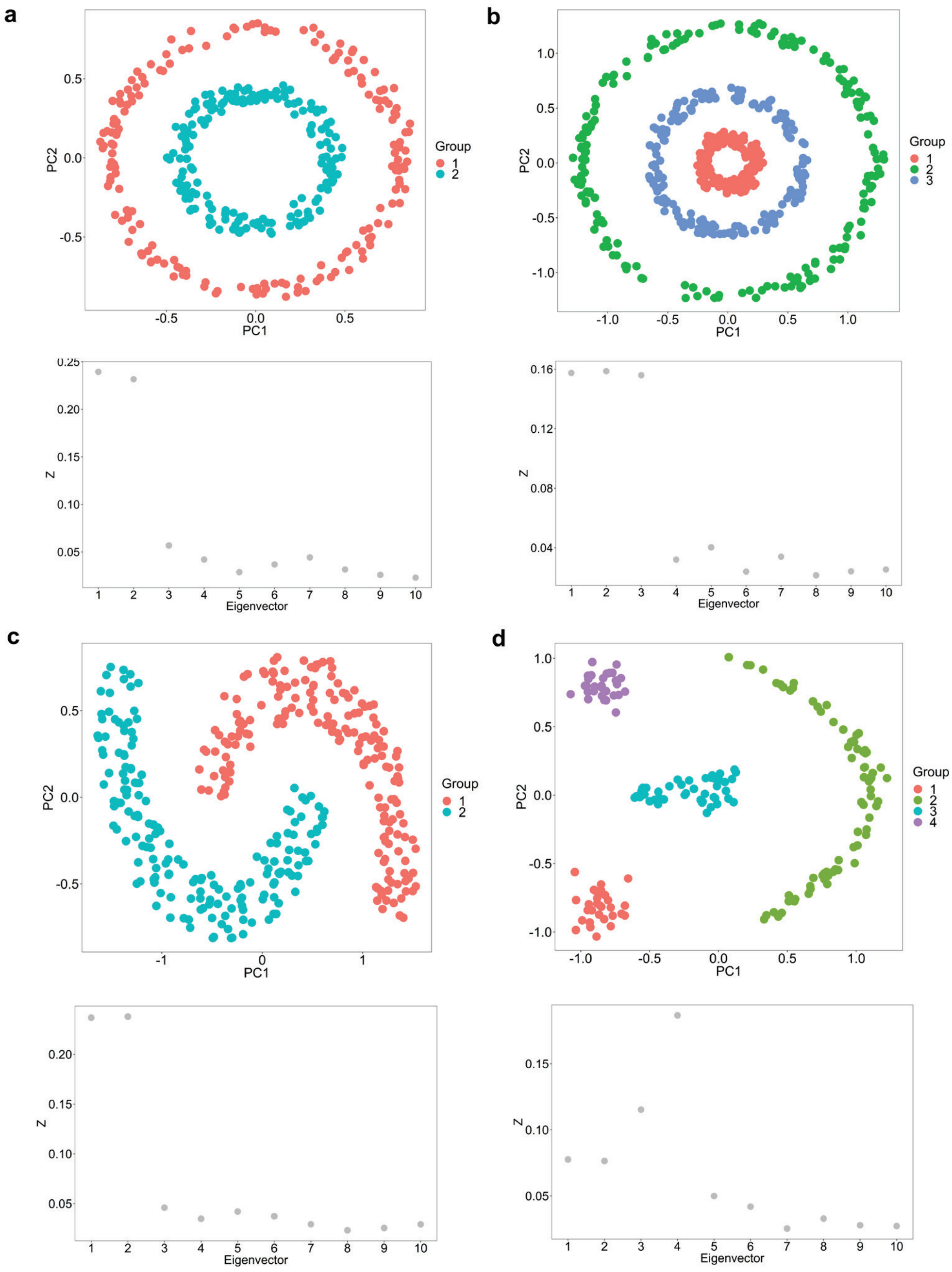

Supplementary Fig. S15. Demonstration of the multimodality gap spectral clustering method in recognising non-Gaussian structures. Z refers to the dip test statistic z_i for the ith eigenvector, where a larger value indicates stronger multimodality. (A) Results using multimodality gap method on a two concentric circle dataset. The optimal K was identified because the gap between two and three is the last substantial one (see methods). (B) Results from analysing a three concentric circle dataset, again the correct K was identified. (C) Results from analysing a two half-moon dataset, the correct K was detected. (D) Results from analysing a four cluster smiley face, the correct K was also found.

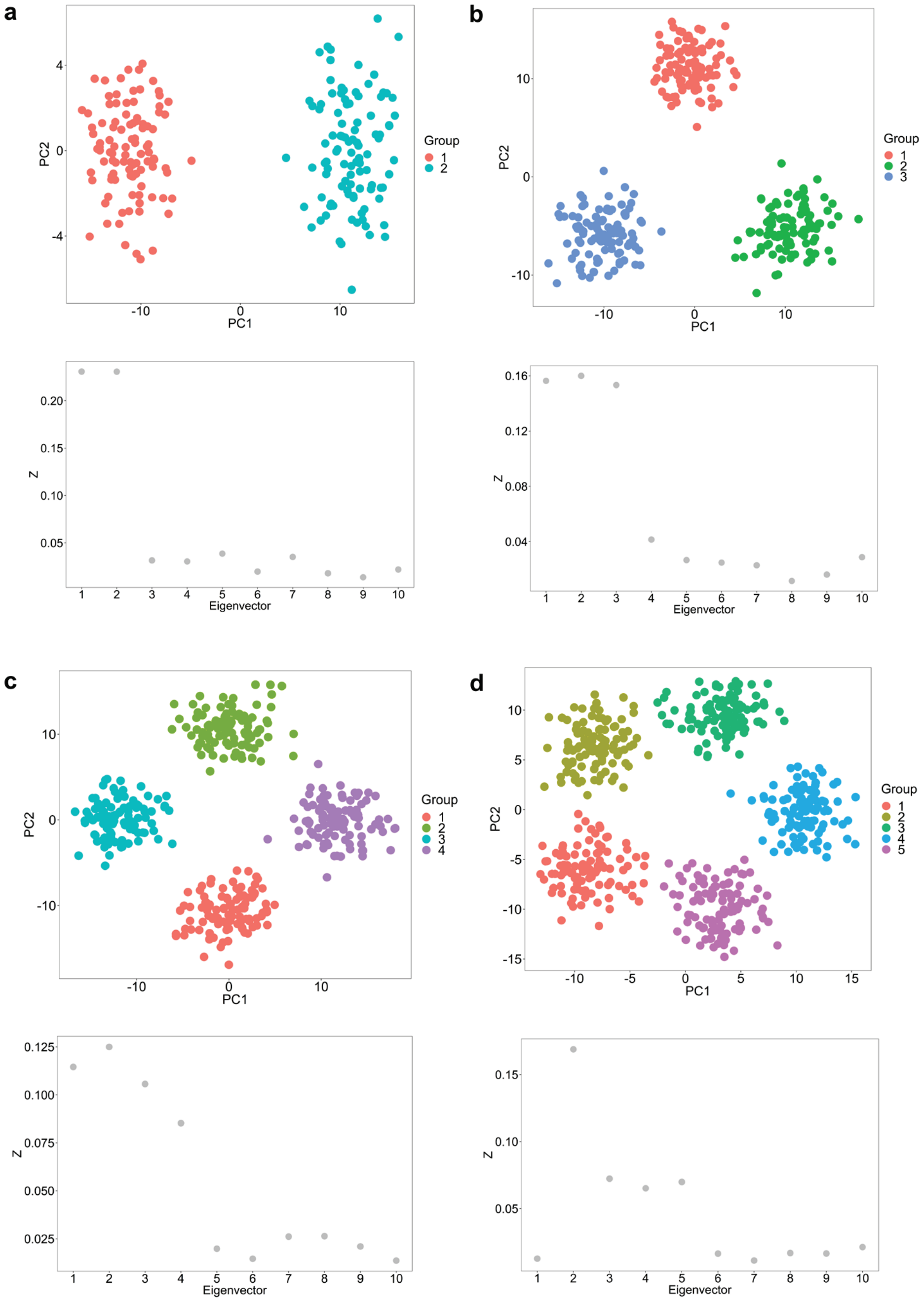

Supplementary Fig. S16. Demonstration of the multimodality gap method in recognising Gaussian clusters. Z refers to the dip test statistic z_i for the ith eigenvector, where a larger value indicates stronger multimodality. (A) Results from using the multimodality gap method on a K=2 Gaussian synthetic dataset. The optimal K of two is identified by the method because the eigenvalue gap between eigenvectors two and three is the last substantial one. (B) Results from using the multimodality gap method on a K=3 Gaussian synthetic dataset, the correct K is identified. (C) Results from using the multimodality gap method on a K=4 Gaussian synthetic dataset, the correct K is identified. (D) Results from using the multimodality gap on a K=5 Gaussian synthetic dataset, if we took the maximum multimodality gap the method fails, therefore we improved the method to find the last substantial gap. This is found by a greedy algorithm described in the Methods.

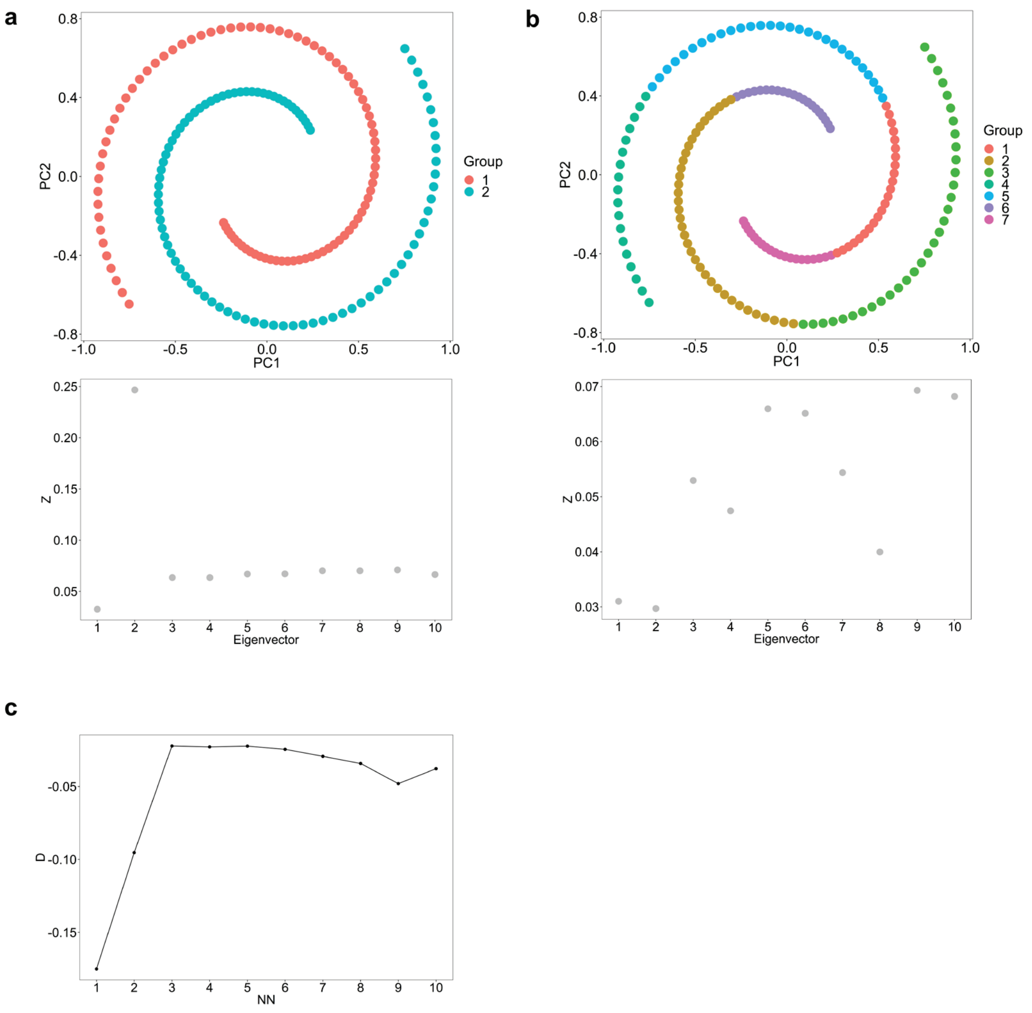

Supplementary Fig. S17. Kernel tuning can improve the multimodality gap method. Z refers to z_i the dip test statistic for the ith eigenvector, where a larger value indicates stronger multimodality. (A) Results from the multimodality Spectral clustering method on spirals with kernel tuning of the K parameter that defines the number of nearest neighbours to use when calculating the local sigma (see Methods). The correct K is found, as the last substantial gap is in-between eigenvectors two and three. (B) Same dataset, but the kernel tuning was not performed, and the correct K was not found. (C) Tuning results from changing the K parameter (NN). D refers to D_{min} the biggest difference between any consecutive elements of z in the set of dip statistics Z for a given value of K. A lower D indicates a bigger difference found for a given K and kernel, therefore indicating a preferred kernel.

Supplementary Table S1. Spectrum TCGA RNA-seq clustering performance compared with other algorithms. P values are from a Cox proportional hazards regression model using a log-rank test to test the significance of the survival time differences between clusters. In brackets next to the p values are the ranks for each dataset (lowest p value corresponds to rank 1). The first final row is the integrated p value using Fisher's method, the second in the sum of the ranks (lower is better). PCPG stands for Pheochromocytoma and Paraganglioma.

Dataset	Datatype	N	Spectrum	PINSplus	M3C	SNF	CLEST
Bladder	mRNA	408	1.29E-05 (1)	0.0013 (3)	0.018 (4)	0.00018 (2)	0.0013 (3)
Brain	mRNA	515	4.03E-22 (1)	0.46 (5)	4.48E-16 (2)	0.038 (4)	4.71E-14 (3)
Breast	mRNA	1093	1.77E-05 (3)	2.60E-06 (2)	9.98E-07 (1)	0.00033 (4)	0.00035 (5)
Kidney	mRNA	533	1.98E-07 (1)	0.4 (5)	0.33 (4)	0.3 (3)	1.34E-06 (2)
PCPG	mRNA	179	0.4	0.56	0.36	0.46	0.43
Skin	mRNA	469	0.00014 (4)	0.00076 (5)	1.14E-07 (1)	8.70E-07 (2)	9.31E-07 (3)
Thyroid	mRNA	501	0.21	0.11	0.084	0.14	0.11
P integrated			2.85E-34	4.55E-08	2.23E-24	1.82E-10	3.60E-25
Rank score			10	20	12	15	16

Supplementary Table S2. Spectrum single cell RNA-seq clustering performance relative to other algorithms. Values used for scoring each algorithm refer to Normalised Mutual Information (NMI) of given cell type labels versus those defined by the clustering algorithm. The bracketed values are the rank for that algorithm relative to the others for each dataset. The first final row corresponds to the summation of the columns NMI values, the second corresponds to the summation of the ranks.

Dataset	N	Spectrum	Seurat	SIMLR	SC3	MUDAN
Camp	777	0.85 (3)	0.87 (1)	0.67 (4)	0.51 (5)	0.86 (2)
Li	561	0.88 (2)	0.86 (3)	0.5 (4)	0.89 (1)	0.86 (3)
Patel	430	0.76 (5)	0.78 (4)	0.79 (3)	0.84 (1)	0.81 (2)
Pollen	301	0.96 (1)	0.86 (4)	0.79 (5)	0.89 (2)	0.87 (3)
Darmanis	466	0.72 (3)	0.78 (1)	0.74 (2)	0.74 (2)	0.7 (4)
Baron	8569	0.84 (1)	0.76 (3)	0.79 (2)	0.76 (3)	0.79 (2)
Muraro	2126	0.88 (1)	0.83 (3)	0.73 (5)	0.86 (2)	0.82 (4)
NMI score		5.89	5.74	5.01	5.49	5.71
Rank NMI score		16	19	25	16	20

Supplementary Table S3. Comparing Spectrum's eigengap procedure with the multimodality gap on multi-omic TCGA data. Values correspond to p values from a Cox proportional hazards regression model using a log-rank test to test the significance of the survival time differences between clusters. The final row is the integrated p value using Fisher's method. PCPG stands for Pheochromocytoma and Paraganglioma.

Dataset	Data	N	Eigengap	Multigap
Bladder	mRNA, miRNA, protein	338	0.0042	0.0028
Brain	mRNA, miRNA, protein	425	3.76E-16	2.39E-16
Breast	mRNA, miRNA, protein	634	1.47E-07	1.99E-07
Kidney	mRNA, miRNA, protein	240	0.91	0.0019
PCPG	mRNA, miRNA, protein	80	0.043	0.7
Skin	mRNA, miRNA, protein	338	0.0014	0.0021
Thryoid	mRNA, miRNA, protein	219	0.049	0.063
			1.07E-22	5.29E-24
